# A cell transcriptomic profile provides insights into adipocytes of porcine mammary gland across development

**DOI:** 10.1186/s40104-023-00926-0

**Published:** 2023-10-08

**Authors:** Yongliang Fan, Long Jin, Zhiping He, Tiantian Wei, Tingting Luo, Jiaman Zhang, Can Liu, Changjiu Dai, Chao A, Yan Liang, Xuan Tao, Xuebin Lv, Yiren Gu, Mingzhou Li

**Affiliations:** 1 State Key Laboratory of Swine and Poultry Breeding Industry, College of Animal Science and Technology, Sichuan Agricultural University, Chengdu, 611130 China; 2 Key Laboratory of Qinghai-Tibetan Plateau Animal Genetic Resource Reservation and Utilization, Southwest Minzu University, Chengdu, 610041 China; 3 Animal Breeding and Genetics Key Laboratory of Sichuan Province, Sichuan Animal Science Academy, Chengdu, 610000 China

**Keywords:** Adipocytes, Cell–cell interaction, Development, Mammary gland, snRNA-seq

## Abstract

**Background:**

Studying the composition and developmental mechanisms in mammary gland is crucial for healthy growth of newborns. The mammary gland is inherently heterogeneous, and its physiological function dependents on the gene expression of multiple cell types. Most studies focused on epithelial cells, disregarding the role of neighboring adipocytes.

**Results:**

Here, we constructed the largest transcriptomic dataset of porcine mammary gland cells thus far. The dataset captured 126,829 high-quality nuclei from physiological mammary glands across five developmental stages (d 90 of gestation, G90; d 0 after lactation, L0; d 20 after lactation, L20; 2 d post natural involution, PI2; 7 d post natural involution, PI7). Seven cell types were identified, including epithelial cells, adipocytes, endothelial cells, fibroblasts cells, immune cells, myoepithelial cells and precursor cells. Our data indicate that mammary glands at different developmental stages have distinct phenotypic and transcriptional signatures. During late gestation (G90), the differentiation and proliferation of adipocytes were inhibited. Meanwhile, partly epithelial cells were completely differentiated. Pseudo-time analysis showed that epithelial cells undergo three stages to achieve lactation, including cellular differentiation, hormone sensing, and metabolic activation. During lactation (L0 and L20), adipocytes area accounts for less than 0.5% of mammary glands. To maintain their own survival, the adipocyte exhibited a poorly differentiated state and a proliferative capacity. Epithelial cells initiate lactation upon hormonal stimulation. After fulfilling lactation mission, their undergo physiological death under high intensity lactation. Interestingly, the physiological dead cells seem to be actively cleared by immune cells via CCL21-ACKR4 pathway. This biological process may be an important mechanism for maintaining homeostasis of the mammary gland. During natural involution (PI2 and PI7), epithelial cell populations dedifferentiate into mesenchymal stem cells to maintain the lactation potential of mammary glands for the next lactation cycle.

**Conclusion:**

The molecular mechanisms of dedifferentiation, proliferation and redifferentiation of adipocytes and epithelial cells were revealed from late pregnancy to natural involution. This cell transcriptomic profile constitutes an essential reference for future studies in the development and remodeling of the mammary gland at different stages.

**Supplementary Information:**

The online version contains supplementary material available at 10.1186/s40104-023-00926-0.

## Background

The mammary gland is an exocrine gland of ectodermal origin, which develops to produce milk for the nourishment of offspring. As a highly dynamic organ, the mammary gland undergoes a limited embryonic development followed by extensive postnatal pubertal development, and further differentiation and tissue remodeling during pregnancy and lactation. The mammary gland is inherently heterogeneous, with various cells performing different functions. The mammary epithelium contains two major cell types, luminal and myoepithelial cells [1, 2], and they are the main components of acini. The lumen of each acinus is hollow with milk secretions during lactation. Milk ejection relies on the contractility of myoepithelial cells [3]. Fibroblasts actively participate in tissue remodeling, synthesizing and secreting collagen, and organizing into bundles in the developing mammary gland [4]. Immune cells participate in and influence branching morphogenesis [5]. Another type of cell, adipocytes, comprise a large portion of the stromal compartment in the adult non-lactating mammary gland, but gradually disappear during pregnancy, freeing up room for the expanding mammary glands [6]. In 2013, Gregor et al. [7] selectively knocked out X-box binding protein 1 (*Xbp1*) of adipocyte in mice mammary gland during lactation, causing adipocytes proliferation and lower milk production. In 2014, Vapola et al. [8] knocked out the peroxisomal membrane protein 2 (*Pxmp2*) gene in adipocytes of the mice mammary gland, which restricts epithelial cells development and duct formation during pregnancy. In 2018, Wang et al. [6] reported that adipocytes from mammary gland can become PDGFRα^+^ preadipocytes and fibroblast-like preadipocytes through dedifferentiation during lactation. In addition, the gene expression level is significantly different between the dedifferentiated cells and the adipocytes in the non-lactating mammary gland. During involution, PDGFRα^+^ preadipocytes will proliferate and differentiate into adipocytes. Although adipocytes play such a critical role in gland development, most studies focused on its epithelial component, leaving the role of the neighboring adipocytes largely unexplored in both physiologic and pathologic conditions [9].

Cell fate decisions are largely based on gene transcription. Therefore, it is particularly critical to identity the cell-types and the gene transcription profile of individual cells to understand mammary gland physiology. Recently, single-cell RNA sequencing (scRNA-seq) emerged as a powerful technique to study complex biological systems at single-cell resolution. In 2017, Bach et al. [10] systematically constructed cell transcriptomic atlas of the epithelial cells from mouse mammary gland across four developmental stages based on scRNA-seq data. The atlas indicated that mammary epithelial cells are not a group that performs a single function, and could be divided into four classes: basal, mature luminal, luminal progenitor, and luminal intermediate cells. Subsequent studies identified immune cells, fibroblasts and endothelial cells in the mammary gland [11–14]. Individual cells display state-specific expression patterns. For instance, principal component analysis (PCA) exposed gene transcription differences in both basal and luminal cells in pregnant and nonpregnant mice mammary gland [15]. Moreover, Brugge and co-workers confirmed age-dependent alterations in gene expression by analyzing epithelial, stromal, and immune cells in mice mammary gland [12]. Remarkably, mammary gland development and function depends on intricate interactions of the functional epithelial cells with local stromal cells [16, 17]. In other words, clarification of cell–cell interaction is required.

Considering the lack of focus on adipocytes in mammary glands and the limitations of scRNA-seq, it is not surprising they are rarely identified in the mammary gland. In detail, cells over 50 μm in diameter are difficult to capture by microfluidic droplet generators, hindering gene transcription profiling of tissue-derived adipocytes by scRNA-seq approaches. Furthermore, not all studies identify the same mammary cell types and most agree that cell subpopulations such as secretory alveolar cells during lactation have been incompletely profiled due to technical difficulties to isolate intact cells during dissociation [10]. Notably, single-nucleus RNA-seq (snRNA-seq) has become instrumental to interrogate oversized cells or in complex tissues that are not easily dissociated. This approach enabled the comprehensive mapping of mammary cell transcription in different physiological states. Regrettably, only one report identified adipocytes in mammary glands of adult human using snRNA-seq as of June 2023 [18], which means that, to date, no gene transcription data from adipocytes of porcine mammary glands is available.

The physiological function of a tissue is not only dependent on the gene expression of its individual cells. The internal spatial organization of these cells is also critically important [19]. However, spatial information of individual cells is lost during tissue dissociation for snRNA-seq sequencing [20, 21]. Contrastingly, spatial transcriptomics (ST), a more recent method, enables the visualization and quantitation of the transcriptome in individual tissue sections, retaining spatial molecular information [22]. However, the spatial expression pattern of adipocytes in healthy porcine mammary glands has not been reported. Thus, we describe the spatial transcriptome profiles of adipocytes in non-lactation mammary gland. Furthermore, this spatial expression profile validates the accuracy of the cell type annotation based on snRNA-seq data.

In this study, we constructed the largest transcriptomic dataset of the porcine mammary gland thus far to reveal its development at d 90 of gestation (G90), d 0 after lactation (L0), d 20 after lactation (L20), 2 d post natural involution (PI2), 7 d post natural involution (PI7). Seven cell types were identified in this dataset, including epithelial cells, adipocytes, endothelial cells, fibroblasts cells, immune cells, myoepithelial cells and precursor cells. Their gene transcription patterns were determined, and an interaction network between adipocytes and other cell groups was constructed at both snRNA-seq and spatial transcriptomics levels. To explore the impact of developmental stages on milk composition, we identified the type and proportion of milk secreted during colostrum and mature milk using scRNA-seq. Cell phenotype analysis showed more abundant macrophages in colostrum than mature milk, which likely explains why colostrum possess innate immune activity. The swine cell atlas here reported will guide future studies in mammary physiology. It also provides insights into the development stage-specific gene expression profiles of epithelial cells and adipocytes.

## Materials and methods

### Sample collection and histological observation

**Fig. 1:**
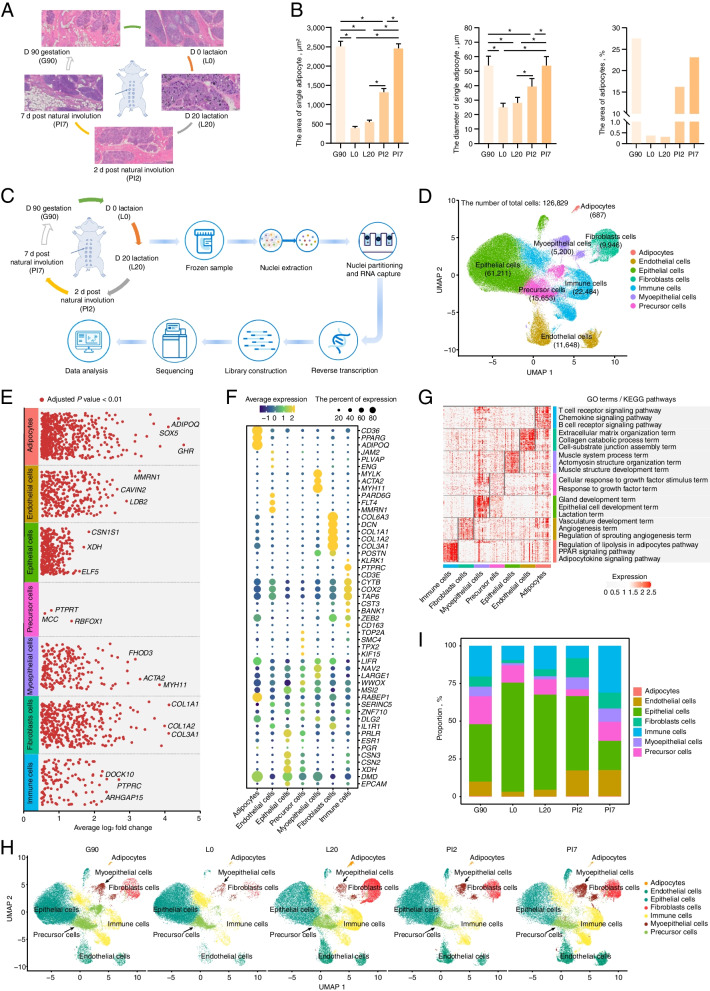
Generation of a stage-specific single-cell atlas of the pig mammary gland. **A** Histological observations of the mammary gland at five developmental stages. **B** Statistical analysis of adipocytes area and size. **C** Schematic workflow for snRNA-seq sequencing. **D** UMAP visualization of all clusters colored by all cell types. Seven cell clusters were identified in the dataset. **E** The DEGs analysis shows upregulated genes (Adjusted *P* value < 0.01) across all seven clusters. **F** Cell type annotation for all clusters is provided in the bubble chart. **G** GO annotation and KEGG pathway analysis of differentially expressed genes in each cell-type. **H** UMAP illustration of cells colored by clusters in separate development stage. **I** Stacked bar plots represent the proportions of nuclei in the mammary gland

Multiparous sows in second breeding cycle were divided into 5 groups [d 90 of gestation (G90); d 0 (L0), d 20 (L20) after lactation; 2 d (PI2) and 7 d (PI7) post natural involution]. Each group has 4 independent biological replicates. First parity sows usually face higher risk and stress response. Thus, we selected the multiparous sows as an experimental animal to ensure the reliability and reproducibility of the results. All pigs were fed well-characterized normal diets, according to the nutritional requirements outlined by the Feeding Standard of Swine (NY/T 65–2004) and published by the Ministry of Agriculture and Rural Affairs of the People’s Republic of China [23]. Sows were humanely euthanized at five developmental stages for collection of the mammary glands (approximately 3 cm^3^) from the third region on the right side (Fig. 1A). Part of fresh tissue was used to prepare frozen sections for histological observation (HE staining, *n* = 4) (G90, L0, L20, PI2 and PI7) and spatial transcriptome sequencing (*n* = 1) (G90 and PI2). Meanwhile, the remaining tissue (*n* = 2) (G90, L0, L20, PI2 and PI7) was collected, immediately snap-frozen in liquid nitrogen, and then transferred to −80 °C until further nuclei isolation. In addition, 15 mL fresh colostrum (L0) and mature milk (L20) were collected for scRNA-seq (*n* = 1) for cellular component analysis.

### Single nuclei/cell RNA sequencing

The snRNA-seq and scRNA-seq were respectively performed for the construction of the transcriptional atlas of the mammary glands and milk cellular components. Nuclei isolation was carried out using GEXSCOPE® Nucleus Separation Solution (Singleron Biotechnologies, Nanjing, China) according to the manufacturer's product manual. Isolated nuclei were resuspended in PBSE to 10^6^ nuclei per 400 μL, filtered through a 40-μm cell strainer, and counted with Trypan blue. The concentration of single nuclei suspension was adjusted to 3–4 × 10^5^ nuclei/mL in PBS. Subsequently, single nuclei/cell suspension was loaded onto a microfluidic chip (GEXSCOPE® Single NucleusRNA-seq Kit/GEXSCOPE™ Single-Cell RNA Library Kit, Singleron Biotechnologies) and snRNA-seq/scRNA-seq libraries were constructed according to the manufacturer’s instructions (Singleron Biotechnologies). Finally, the resulting snRNA-seq/scRNA-seq libraries were sequenced on an Illumina HiSeq × 10 instrument with 150 bp paired end reads.

### Data processing and cell-type annotation

Sequencing reads were processed using the CeleScope v1.1.7 pipeline (Singleron). Then, raw reads were aligned to the *Sscrofa11.1* reference genome, generating count matrices. To remove low-quality droplets, we excluded any nuclei or cells expressing less than 300 genes and more than 25% mitochondrial genes. After filtering, 126,829 nuclei were reserved and used for dimension-reduction and clustering. The normalization and scale of all gene expression values were performed by NormalizeData and ScaleData function. Principal component analysis (PCA) relied on the top 2000 variable genes screed by FindVariableFeatures. Next, integration of snRNA-seq and scRNA-seq data was respectively done using the harmony package (https://github.com/immunogenomics/harmony) to control for batch effects when integrating data from different development samples. For dimensionality reduction, the top 15 principal components were selected to calculate 2D dimensional reductions by Uniform Manifold Approximation and Projection for Dimension Reduction (UMAP) for all sequencing libraries based on an elbow approach. Finally, cell clusters were identified using the Louvain algorithm at a resolution of 0.3, implemented by the FindCluster function of Seurat (v4.3.0).

The clusters were partitioned into distinct cell types and annotated by the expression of known marker genes. The expression signatures of cell‐type‐specific genes were detected using the “FindAllMarkers” function. The criteria to identify cell‐type‐specific genes were set as follows: absolute log_2_ fold change (FC) ≥ 1 and the minimum cell population fraction in either of the two populations was 0.25. The expression pattern of each marker gene was visualized by applying the “DotPlot” function in Seurat.

### Gene function analysis

To reveal gene transcription dynamics of adipocytes and epithelial cells, we detect time-series expression profiles of differentially expressed genes (DEGs) between adjacent development stages using Mfuzz algorithm. DEGs were identified using FindMarkers function in Seurat (log_2_FC > 1, *P* < 0.05). The Kyoto Encyclopedia of Genes and Genomes (KEGG) and Gene Ontology (GO) enrichments were conducted to identify the main function of the DEGs having the same expression trend using the ClusterProfiler v4.0.2 package.

In addition, we identified DEGs (log_2_FC > 1, *P* < 0.05) between any two adjacent developmental stages of the annotated seven cell types. To clarify their biological function, GO annotation and KEGG pathway analysis were performed, generating 28 sets of significant GO terms and KEGG pathways. We compared four sets of significant terms and pathways in each cell type, and visualized the results using Venn Diagrams. Another group of Venn diagrams displayed the intersection of GO terms or KEGG pathways and were annotated in the seven type cells in different physiological processes.

### Pseudotimeanalysis

To characterize the physiological state of epithelial cells and adipocytes in G90 and PI2, we calculate their differentiation trajectory based on default parameters of DDRTree method in Monocle2 package. In detail, normalized unique molecular identifier (UMI) count was fed as the input for Monocle2. Genes with a residual greater than 1× the estimated mean–variance split, were identified as high-dispersion genes using the “estimateDispersions” method. After running the “setOrderingFilter” function, dimensionality reduction was applied to the data with the default parameter of DDRTree method [24]. The trajectory was visualized by plot_cell_trajectory function. Furthermore, branch analysis was performed by branched expression analysis modeling (BEAM), and visualized via the plot_genes_branched_heatmap function [25].

### Cell–cell interactions

The high-confidence ligand (L)—receptor (R) interactions were performed to investigate the interaction between mammary gland cells in five development stages using the iTALK package [26]. Expressed genes were selected for L-R interaction analysis according to the following criteria: (1) the top 20 highly expressed genes and (2) marker genes in corresponding clusters.

### Spatial transcriptome sequencing of mammary gland

Spatial expression of mammary gland during G90 and PI2 was performed to evaluate cell-type annotation accuracy. In detail, the 10 μm frozen tissue sections were placed on one of the Visium gene expression slide capture areas in a slide. The RNA quality of mammary glands was assessed by Agilent 2100, and RNA integrity number (RIN) of tissues greater than 7 were used for Visium spatial gene expression experiments. The Visium Spatial Gene Expression Slide & Reagent kit (10 × Genomics) was used to construct sequencing libraries according to the Visium Spatial Gene Expression User Guide (CG000239, 10 × Genomics). Tissue permeabilization was optimized during the tissue optimization procedure. Reverse transcription experiment and sequencing libraries were then prepared following the manufacturer’s protocol. Sequencing was performed with a Novaseq PE150 platform according to the manufacturer’s instructions (Illumina) at an average depth of 300 million read-pairs per sample.

We used in-house script to perform basic statistics of raw data, and evaluate the data quality and GC content along the sequencing cycles. Raw FASTQ files and histology images were processed by sample with the Space Ranger (version spaceranger-1.2.0, 10 × Genomics) software with default parameters. The filtered gene-spots matrix and the fiducial-aligned low-resolution image was used for down-streaming data analyses.

Reads were demultiplexed using Space Ranger software v.1.0.0 (10 × Genomics) and annotated with the reference genome Sscrofa11.1. Subsequently, the generated count matrices were loaded into Seurat environment, and the data were normalized (using the ‘SCTransform’ function in Seurat for independent tissue sections), reduced and visualized. SPOTlight analysis was performed for deconvolution analysis as Elosua-Bayes et al. reported [27]. In brief, the proportion of signature of the selected snRNA-seq cell type is equal to the sum of the proportions of each cell type in different regions, divided by the sum of the proportions of that cell type in all spots.

### Statistical analysis

The statistical analysis of the area and diameter of adipocytes were performed using GraphPad Prism 9 (GraphPad, San Diego, CA, USA) and one-way ANOVA. Data were presented as the mean ± standard deviation (SD), and *P* < 0.05 (*) indicated a significant difference.

### Data availability

Sequencing data were deposited in the Gene Expression Omnibus (GEO) with the accession code GSE227425.

## Results

### Generation of a development stage-specific single-cell atlas of mammary gland

We collected mammary glands of ten female pigs across five developmental stages, including d 90 of gestation (G90); d 0 (L0) and 20 (L20) after lactation; 2 d (PI2) and 7 d (PI7) post natural involution for histological observations (Fig. 1A). Mammary gland sections stained with HE indicated that acini appeared in late gestation (G90). In addition, the mammary gland went through remarkable morpho-functional changes in its adipocytic components, i.e., both area and size of adipocytes were greater in non-lactation than lactation stages (Fig. 1B and Table S1).

To understand the tissue composition and gene-expression dynamics, we generated snRNA-seq profiles from 10 mammary glands across the five developmental stages (Fig. 1C). After quality control, a transcriptomic dataset with 126,829 high-quality nuclei were retained, with numbers ranging from 18,693 in the d 90 gestation group to 9,331 in the 2 d post natural involution group (Fig. S1 and Table S2). On average, we detected 2,542 UMIs and 1,395 genes per nuclei. Upon batch effect correction, the 126,829 nuclei separated into multiple clusters (Fig. 1D) using UMAP [28]. Seven cell clusters were defined according to the expression levels of specific markers (Fig. 1E–F and Table S3) including adipocytes, epithelial, fibroblasts, endothelial, myoepithelial, immune and precursor cells (Fig. 1D). For instance, adiponectin (*ADIPOQ*) and epithelial cell adhesion molecule (*EPCAM*) expression subdivide adipocytes and epithelial cells from mammary glands, respectively. The assigned cell types were further confirmed by function analysis of gene sets identified by “FindAllMakers” function in Seurat [29] (Fig. 1G). Unsurprisingly, cellular functions derived from shared gene annotations were associated with phenotypic similarity. The biological functions of the gene sets were linked with the cell types, such as the gland development term, the epithelial cell development term and the lactation term which were only significantly enriched in the annotated epithelial cell. Similarly, the regulation of lipolysis in adipocytes pathway, the PPAR signaling pathway and the adipocytokine signaling pathway were significantly enriched in the annotated adipocytes. This provided further evidence of cell type identification accuracy.

The total number of each cell type ranged from 61,211 (48.26%) for epithelial cells, to 687 (0.54%) for adipocytes in the mammary gland (Fig. 1H and Table S4). In addition, cell-type composition dynamics changed in mammary glands during different developmental stages (Fig. 1I). Globally, adipocytes constituted between 0.34% and 0.09% of all cells present, with a higher proportion in non-lactation period compared with lactation. This is consistent with our phenotypic results (Fig. 1B) and previous study [30]. Conversely, the proportion of epithelial cells, varying from 72.43% to 19.37%, was higher in lactation versus non-lactation period, gradually decreasing gradually with mammary gland remodeling. In addition, the ratio of fibroblasts, endothelial and myoepithelial cells was greater in non-lactation period rather than lactation.

### Gene expression patterns of epithelial cells across five developmental stages

**Fig. 2:**
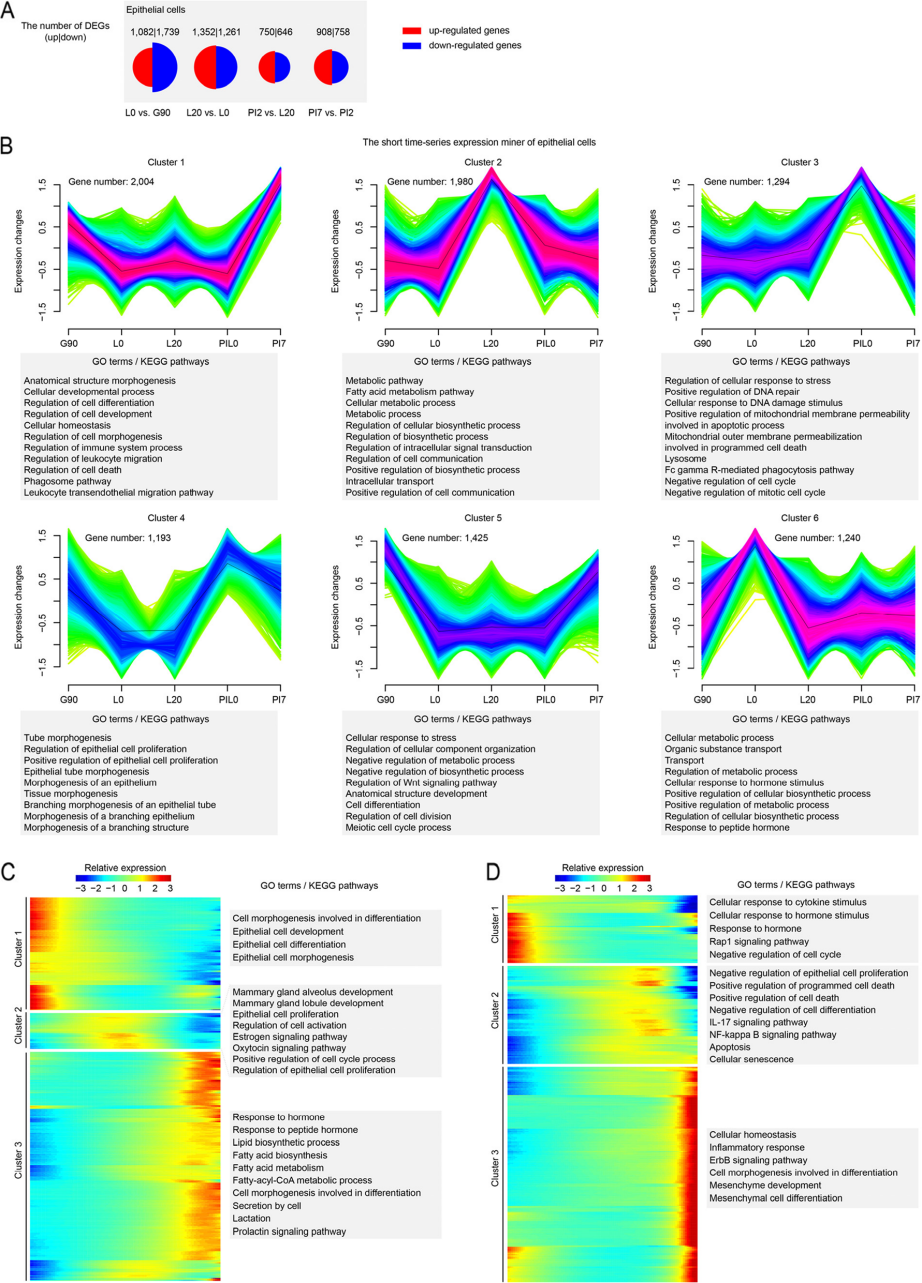
Gene expression patterns of epithelial cells at different developmental stages. **A** Proportional Area Chart (Half Circle). Two groups of half circles indicate two DEGs sets of epithelial cells, and the areas represent the number of DEGs. **B** Fuzzy clustering of expression data at five developmental stages. Purple or red colored lines correspond to genes with high membership value, and *y* axis represents the normalized expression value from the Mfuzz result. **C** Monocle trajectory inference traces a path of pesudotime and group types. **D** The heatmap reveals the relative gene expression level of 3 clusters at 2 branches based on branched expression analysis modeling, combined with the GO/KEGG enriched items for each cluster

To unveil the gene transcription dynamics of epithelial cells across adjacent developmental stages, we identified differentially expressed genes (DEGs) using the FindMarkers function in Seurat (Fig. 2A and Table S5). The time-series gene expression profiling of epithelial cells exhibits six time-dependent expression patterns (Fig. 2B). During late pregnancy (G90), the main functions of highly expressed genes (cluster 5) are regulation of cell division and cell differentiation. After deliver (L0), specific gene-expression (cluster 6) regulated by hormone stimulation initiate lactation. As the newborn grows (L20), the biosynthetic process (cluster 2) is activated in maternal mammary epithelial cells. Upon cessation of suckling by the offspring, the involution of the mammary gland is initiated. In early natural involution (PI2), most of highly expressed genes in epithelial cells (cluster 3) participated in apoptotic and cell proliferation inhibition to allow space for other cell types. At 7 d post natural involution (PI7), the immune mechanism is activated.

Although epithelial cells are captured from the same developmental stage, they are still in different physiological states. Monocle provides a convenient way to screen all pseudotime-dependent genes and identify genes following similar kinetic trends. In late pregnancy (G90), the pseudotime analysis shows three cell subsets with similar gene expression patterns (Fig. 2C). After function analysis, cells from the three subsets performed different functions according to pseudotime. In cell cluster 1, highly expressed genes are mainly enriched in terms or pathways related to cell differentiation, development and morphogenesis. In cell cluster 2, genes participated in hormone signal and the development process of mammary gland, alveolus and lobules. In cell cluster 3, high expression genes promoted lactation and substance synthesis in epithelial cells. Similarly, three cell clusters were identified in early natural involution (PI2) (Fig. 2D). The pseudotime series analysis reveals three physiological states of these cells: cessation of proliferation in response to hormone stimulation (Rap1 signaling pathway), programmed cell death (IL-17 signaling pathway and NF-kappa B signaling pathway), and dedifferentiation into mesenchymal-like cells (mesenchymal cell differentiation).

**Fig. 3:**
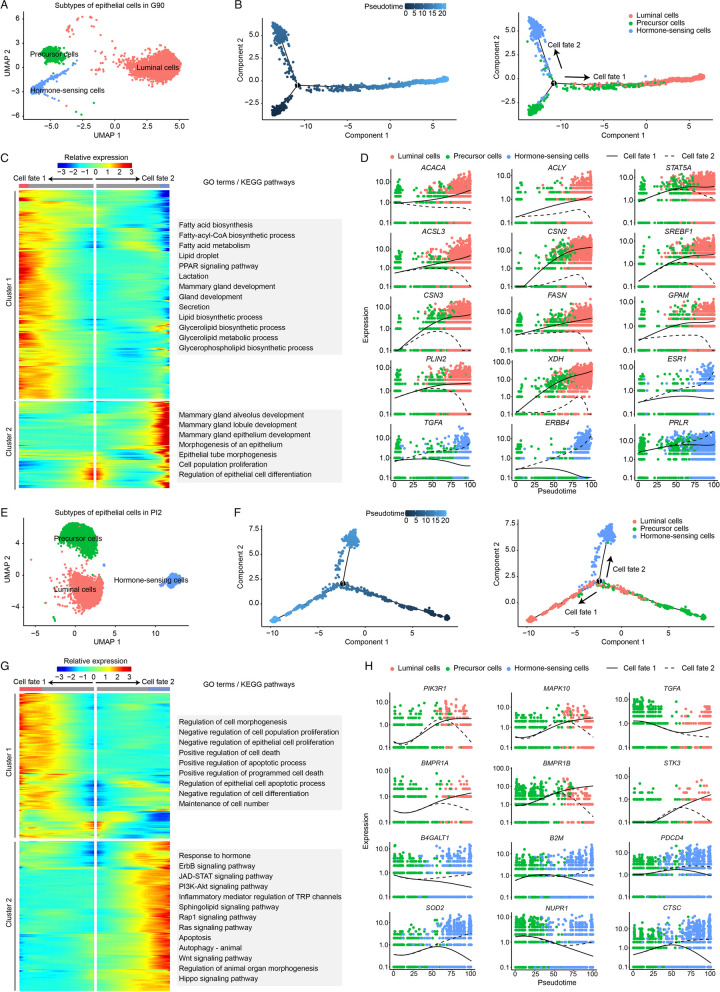
Subtype classification of epithelial cells in G90 (**A**) and PI2 (**E**). Monocle trajectory inference traces a path of pseudotime of epithelial cells in G90 (**B**) and PI2 (**F**). The heatmap reveals the relative gene expression of 3 clusters at 2 branches based on branched expression analysis modeling in G90 (**C**) and PI2 (**G**), combined with the GO/KEGG enriched items of each cluster. Visualization of the transition of highly expressed genes in pseudotime ordering of epithelial cells in G90 (**D**) and PI2 (**H**)

To characterize the physiological state of epithelial cells in G90 and PI2, we used Monocle2 package to calculate the differentiation trajectory of these cells. According to known marker genes [31–33], cells were classified as epithelial precursor cells (*ALDH1A3*, *CD14* and *KIT*), luminal epithelial cells (*CSN2* and *LALBA*) and hormone-sensing epithelial cells (*ESR1*, *PRLR* and *PGR*) (Fig. 3A and Fig. 3E). Cell differentiation trajectories indicate that hormone-sensing cells and luminal cells originate from epithelial precursor cells (Fig. 3B and Fig. 3F).

In late pregnancy (G90), gene function analysis revealed hormone receptor genes are activated in hormone-sensing epithelial cells, including erb-b2 receptor tyrosine kinase 4 (*ERBB4*), transforming growth factor alpha (*TGFA*) and estrogen receptor 1 (*ESR1*) (Fig. 3C–D). Functional activation of luminal cells depended on Acetyl-CoA carboxylase (*ACACA*), fatty acid synthase (*FASN*) and *ACLY* (Fig. 3C–D).

In early natural involution (PI2), the hormone-sensing epithelial cells received hormone stimulation [phosphoinositide-3-kinase regulatory subunit 1 (*PIK3R1*), mitogen-activated protein kinase 10 (*MAPK10*) and *TGFA*] and activate apoptosis related terms and pathways (*BMPR1A*, *BMPR1B* and *STK3*). At the same time, luminal epithelial cells activated the positive regulation of programmed cell death term [superoxide dismutase 2 (*SOD2*), cathepsin C (*CTSC*) and beta-1,4-galactosyltransferase 1 (*B4GALT1*) and the regulation of epithelial cell apoptotic process pathway (beta-2-microglobulin (*B2M*), nuclear protein 1, transcriptional regulator (*NUPR1*) and programmed cell death 4 (*PDCD4*)] (Fig. 3G–H).

### Gene expression patterns of adipocytes across five developmental stages

**Fig. 4:**
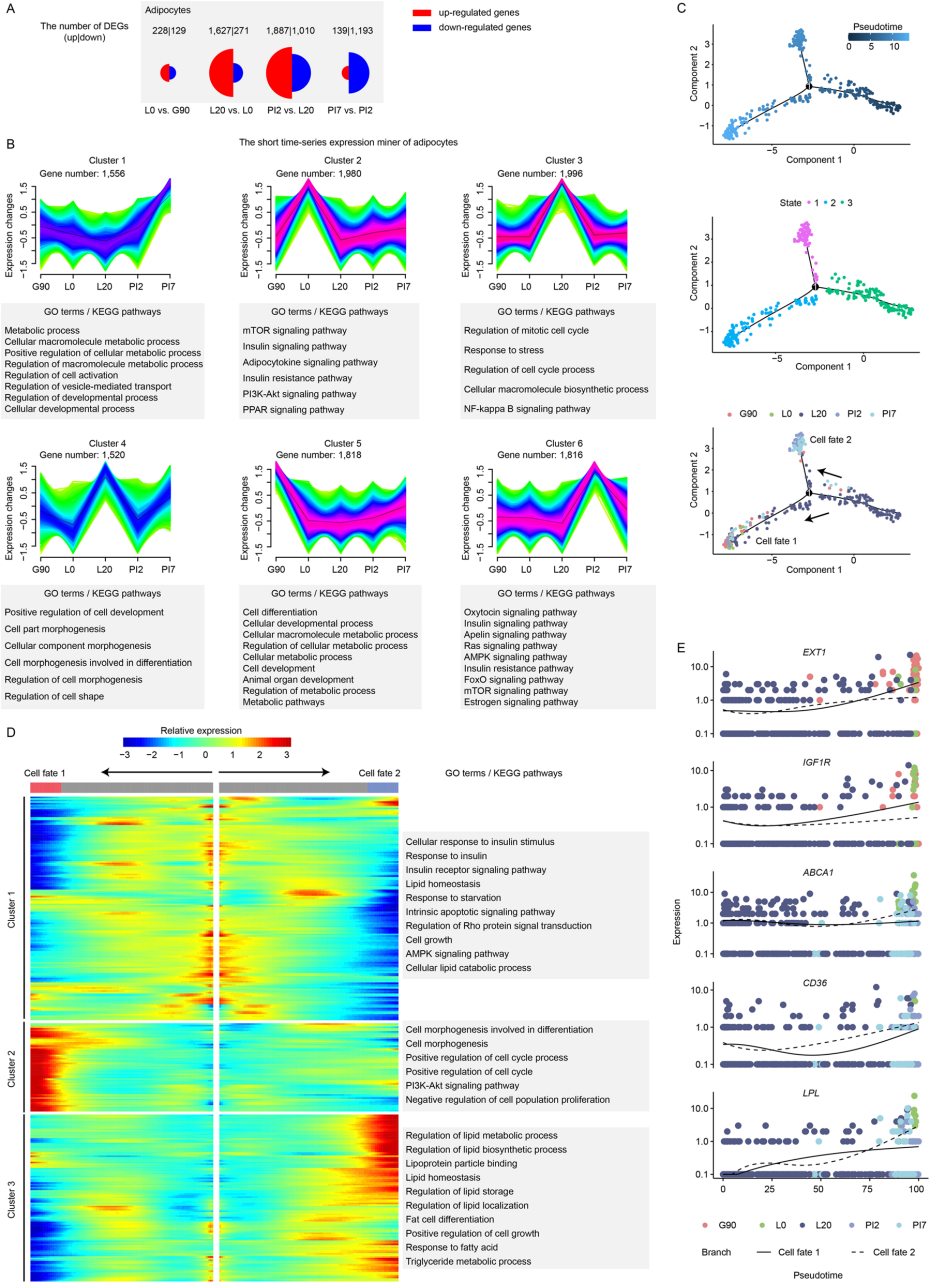
Gene expression patterns of adipocytes at different developmental stages. **A** Proportional Area Chart (Half Circle). Two groups of half circles indicate two DEGs sets of adipocytes, and the areas represent the number of DEGs. **B** Fuzzy clustering of expression data at five developmental points. Purple or red colored lines correspond to genes with high membership value, and *y* axis represents the normalized expression value from the Mfuzz result. **C–D** The heatmap reveals the relative gene expression level of adipocytes in G90 (**C**) and PI2 (**D**), combined with the GO/KEGG enriched items of each cluster. **E** Visualization of the transition of highly expressed genes in pseudotime ordering of adipocytes at different developmental stages

To unveil gene transcription dynamics of adipocytes across adjacent developmental stages, we identify DEGs using the FindMarkers function in Seurat (Fig. 4A and Table S5). Based on DEGs identified from adipocytes, we employed Mfuzz algorithm to detect time-series expression profiles of genes across developmental stages. Six time-dependent expression patterns were characterized in adipocytes and investigated for their biological significance (Fig. 4B). The DEGs of adipocytes in cluster 1 and 5 show high expression levels during non-lactation. The function of these genes is significantly enriched in cellular development and macromolecule metabolic processes term. This is consistent with the results of both our phenotypic data (Fig. 1A–B) and previous research [6]. The DEGs in cluster 2 are specific expression at colostrum stage (L0) versus the other four stages. These DEGs are mainly involved in the response to hormone and adipocytokine. In cluster 3 and cluster 4, the DEGs are highly expressed at L20, and sustain adipocytes survival. This correlates a compression of the adipocyte space during lactation. Highly expressed genes in cluster 6 are mostly involved in the response to hormone stimulus and cell proliferation during early natural involution (PI2).

In many biological processes, cell growth, differentiation and development do not progress in perfect synchrony. Single-cell expression studies of cell differentiation often capture cells distributed across the entire process. To understand the gene gradient expression at different developmental stages, we applied pseudotime and trajectory analysis for adipocytes using Monocle 2. In late pregnancy (G90), the majority of adipocytes are in a highly differentiated state. When the living space is occupied by other cell types (Fig. 1A), adipocytes gradually dedifferentiate during the onset and maintenance of lactation (Fig. 4C). During lactation, highly expressed genes regulate cell morphogenesis and keep adipocytes at undifferentiated state. These highly expressed genes are significantly enriched in the canonical insulin receptor signaling pathway and AMPK signaling pathway (Fig. 4D). In the two pathways, we found three key genes, ZFP36 ring finger protein like 1 (*ZFP36L1*), forkhead box O1 (*FOXO1*), and lipid phosphate phosphohydrolase 1 (*LPIN1*).

In natural involution, the highly expressed genes regulate adipocytes redifferentiation and lipid metabolism (the fat cell differentiation term and the triglyceride metabolic process term) (Fig. 4D). The pseudo time series analysis indicated that adipocytes displayed a mature state at the PI2 and PI7 stages, with terms related to adipocyte substance synthesis, including the regulation of lipid storage term (CD36 molecule, *CD36*), the response to fatty acid term (lipoprotein lipase, *LPL*), and the triglyceride metabolic process term (ATP binding cassette subfamily A member 1, *ABCA1*) (Fig. 4E).

### Cell–cell interactions

**Fig. 5:**
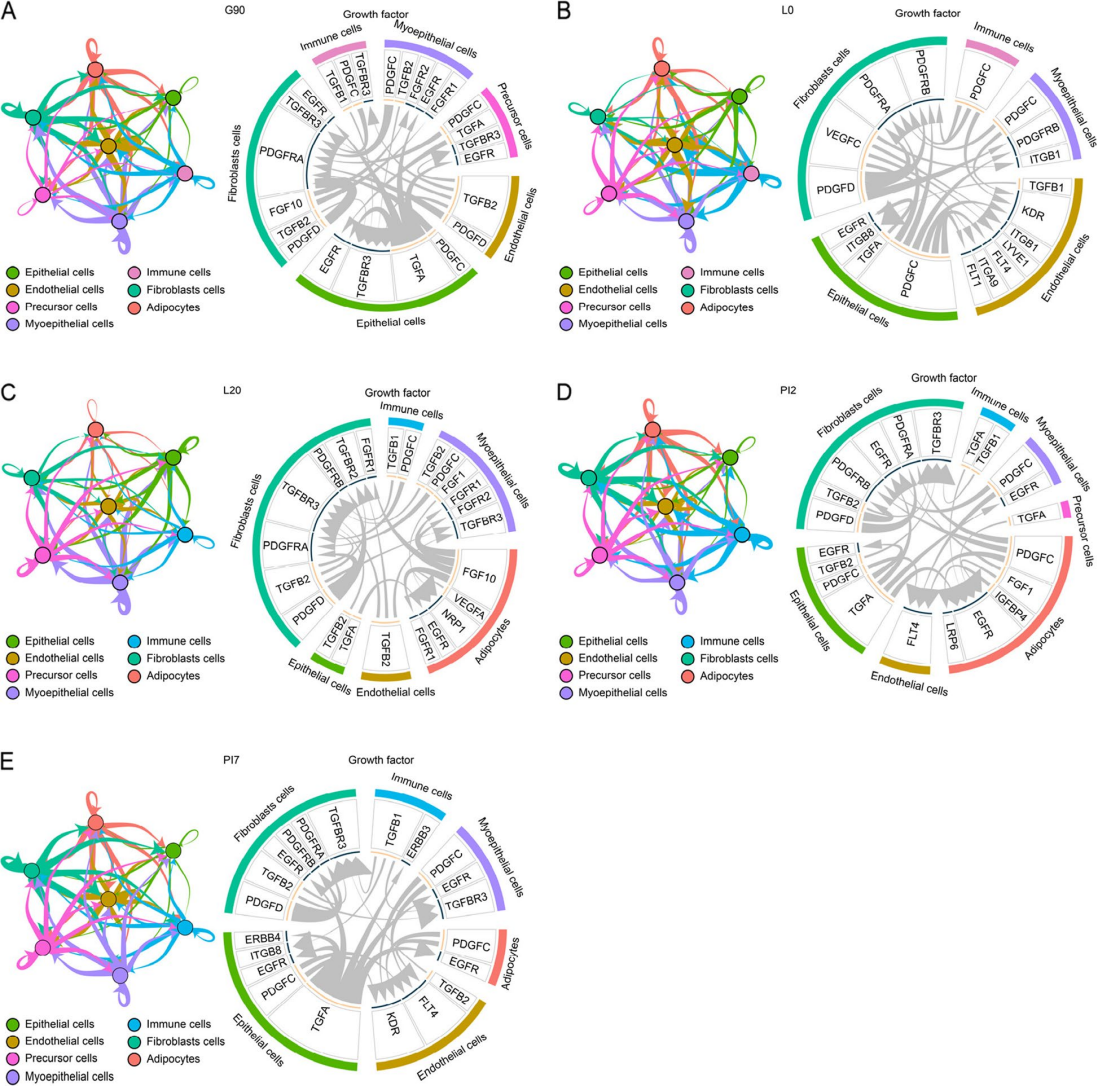
The typical growth factor type ligand-receptor interactions predicted by iTALK between any two cell types. The network plot showed ligand-receptor interactions detected between each two different cell types. In the network, every node showed a cell type, and the thickness of the arrow lines represented the number of ligand-receptor interactions. The arrows labeled the forward (from signaling cell to target cell) and backward signals. The circos plot displayed the names of each ligand-receptor gene pair and the direction. The outside ring of circos plot exhibited cell types, and the inside ring of circos plot exhibited the details of each interaction ligand-receptor pair. The lines inside the circos plot indicated the relative signal strength of the ligand and receptor. The arrow indicated the receptor

Many cell types of the mammary gland contribute to its structure, development, and ultimate function in a dynamic and reciprocal fashion. As proof of principle of the application of this dataset for describing mammary gland cell–cell interactions, we next detected the distribution of cytokine, growth factors and other receptors across the seven type cells. The number of paired ligand-receptor (L-R) interactions showed in the network plots reveals the interactions between each two different cell types and within the same cell type. The Circos plots display the top 20 L-R pairs (Fig. 5 and Fig. S2). In terms of growth factors, the interactions between epithelial cells and other cells are most common during d 90 gestation than the other four stages. Aside from adipocytes, epithelial cells strongly bind endogenous and exogenous growth factor ligands secreted by other cell types. Interactive networks displayed the L-R pairs of growth factors which promoted epithelial cell development, these were mainly driven by TGFB2-TGFBR3, TGFA-EGFR and TGFBR1-TGFBR3 pathways (Fig. 5 and Fig. S2). At the same time, endothelial cells promote the growth of other five cell types, except adipocytes, by secreting TGFB2 and PDGFD ligand. Meanwhile adipocytes showed weak interactions with other cells. Interestingly, the top 20 cell interactions suggest that the interaction between precursor cells and other cell populations only occurs in the transition state of non-lactation and lactation, that is, G90 and PI2 periods (Fig. 5A and D).

At the onset of lactation (L0), the cytokine-type L-R pairs (CXCL12-ITGB1) connects endothelial cells with other cells, except adipocytes and precursor cells. Another important feature of the L0 period, is that the epithelial, myoepithelial and immune cells communicate with endothelial cells via PDGFC-KDR pathway (Fig. 5 and Fig. S2). Fibroblasts secret VEGFC ligand to combined with FLT1, FLT4, LYVE1, ITGA9 and ITGB1 receptors from endothelial cells. At mature milk stage (L20), the cell–cell interactions show that the cytokine ligands secreted by endothelial cells (CCL5, CCL21) and myoepithelial cells (CCL2) activate the adipocyte surface receptor ACKR4 (Fig. 5C and Fig. S2). In addition, two autocrine pathways (FGF10-FGFR1 and ADIPOQ-ADIPOR2) were activated during this period.

In 2 d post natural involution (PI2), adipocytes frequently received cytokine signals from other cell types (Fig. 5D and Fig. S2). Notably, different from mature milk stage, the autocrine pathways maintained the adipocyte proliferation through FGF1-EGFR and IGFBP4-LRP6 pairs at PI2, rather than FGF10-FGFR1 pairs (Fig. 5 and Fig. S2). In epithelial cells, the positive regulation of programmed cell death term was activated. All mammary cells sent out the demand for proliferation and differentiation to immune cells via IL34-CSF1R pairs. In post natural involution (PI2 and PI7), epithelial cells stimulated myoepithelial cell growth through the growth factor type ligand-receptor (TGFA-EGFR) pathway during early natural involution (Fig. 5 and Fig. S2). In PI7, TGFA regulated the proliferation of epithelial cells through an autocrine signaling pathway (TGFA-ERBB4).

### Epithelial cells and adipocytes annotated in situ with precise spatial resolution

**Fig. 6:**
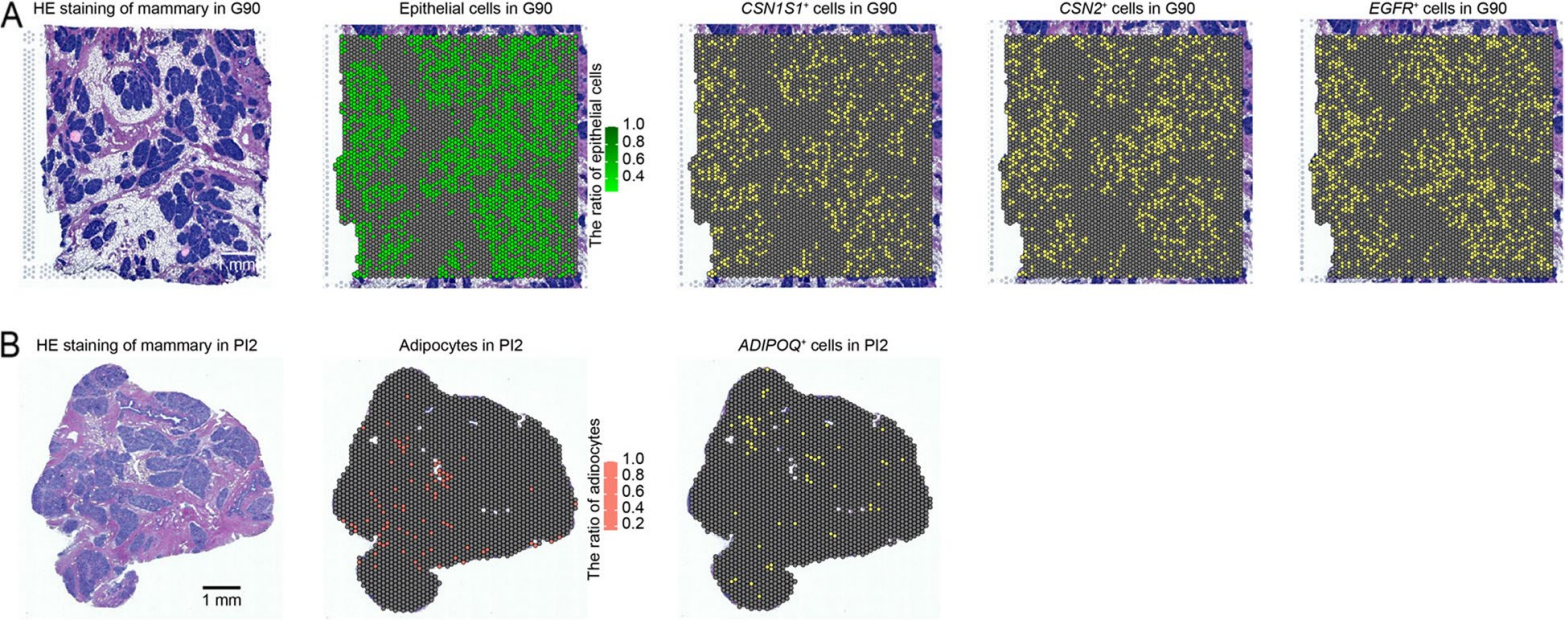
Spatial transcriptome profiles of the mammary gland at G90 and PI2. The epithelial cells and adipocytes were annotated according to gene-makers. Spatial plots showing the expressions of *CSN1S1*, *CSN2*, *EGFR* and *ADIPOQ* genes

Mature acini, the basic unit of galactosis, are observed in the G90, whose main component is mammary epithelial cells. Acinar maturation indicates that the mammary gland has been fully prepared for lactation during G90. Additionally, casein alpha s1 (*CSN1S1*) and casein beta (*CSN2*) are the key genes for mammary epithelial cells participation in lactation. However, snRNA-seq loses spatial information of the expression profiles of *CSN1S1* and *CSN2*. Thus, we verified that the two genes were specifically expressed in the acinar region using spatial transcription sequencing (Fig. 6A and Fig. S3). In detail, we integrated the snRNA-seq and spatial datasets based on SPOTlight with a deconvolutional procedure, generating the spatial distribution of sequenced cells. The spots with *CSN1S1* and *CSN2*-specific expression are largely overlapping with the region annotated as epithelial cells. We found that *EGFR* expression was a specific expression marker in epithelial cells from G90 (Fig. 6A), and repopulation of adipocytes a distinctive feature of natural involution (PI2) (Fig. 1A). The spatial distribution of adipocytes annotated by SPOTlight showed a consistent spatial colocalization of adipocytes in HE-stained section. *ADIPOQ* is a marker of adipocytes that encodes a protein hormone, adiponectin, involved in the regulation and inhibition of lipogenesis and the stimulation of fatty acid oxidation. Its spatial expression pattern also supports the annotation of snRNA-seq data (Fig. 6B and Fig. S3).

### Single-cell sequencing of milk cells from colostrum and mature milk

**Fig. 7:**
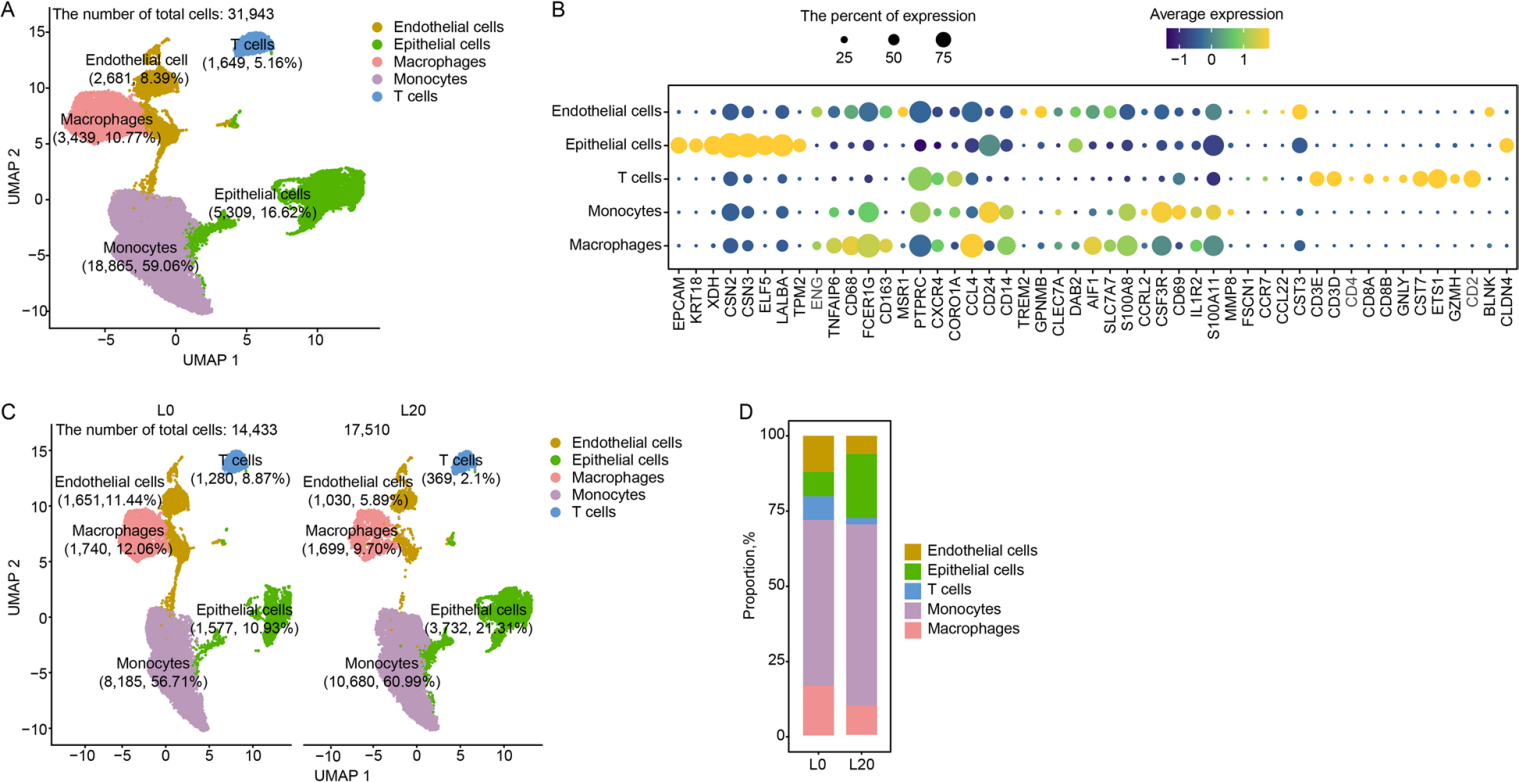
The immune cellular composition of colostrum and mature milk. **A** UMAP plot of five clusters from all sequenced milk cells. **B** Maker genes of the five cell types. **C** UMAP plot of endothelial cells, epithelial cells, macrophages, monocytes and T cells in two samples. **D** The proportion of bar plot of five clusters originating from two samples

The lactation stage affects the cellular component of milk [34]. To explore this effect, we collected fresh colostrum (L0) and mature milk (L20) for scRNA-seq. A total of 31,943 high-quality cells were captured from colostrum and mature milk, including endothelial cells, epithelial cells, macrophages, monocytes and T cells (Fig. 7A). The cell-type classification showed a higher number of macrophages in colostrum (12.06%) versus mature milk (9.70%) (Fig. 7C and D).

## Discussion

The mammary gland provides essential nutrients to the suckling infant [35, 36]. In most mammals, mammary gland morphogenesis begins at embryonic period. After birth, it undergoes three successive stages: puberty, pregnancy and lactation, and natural involution [31, 37, 38]. To date, the adipocyte’s transcriptome profile in swine mammary glands has not been reported. Therefore, the mechanisms by which adipocytes regulate porcine mammary developments and participate in remodeling remains to be elucidated. Here, we measured the gene expression of swine mammary glands across five development stages using snRNA-seq, capturing porcine mammary adipocytes. In this dataset, another six cell types were also annotated; epithelial, fibroblasts, endothelial, myoepithelial, immune and precursor cells. In addition, histological observations revealed a dynamic change in the area and size of the adipocytes through mammary gland development, and indicated that acini appeared in late gestation (G90), which implies cells in mammary gland are preparing for lactation at this developmental stage [39, 40].

In late pregnancy (G90), the majority of adipocytes are highly differentiated, with minimal communications with other cells. Such minimal communications means that adipocytes are less regulated by growth factors than the other six cell groups in G90. We believe this is the reason for the lower adipocyte proportion in the subsequent L0 period. Functional analysis of gene clusters that control cell fate unveiled the activation of GO terms negatively regulated to cell morphogenesis and cell proliferation. This may be the driving force for decreased fat cell volume and number during lactation. In the activated terms and pathways, we found a key gene, *IGF1R*, which regulates cell size through c-Myc family members and plays an important role in the process of cell dedifferentiation, consistent with previous studies [41–43].

Another characteristic event of G90 is that the lactation function of epithelial cells is improved, as specifically shown by the activation of genes related to cell division and differentiation. Most epithelial cells undergo three stages to achieve lactation functions, namely, cellular differentiation, hormone sensing, and metabolic activation. Functional gene analysis revealed that hormone-sensing epithelial cells promote mammary gland development by activated hormone receptor genes, such as *ERBB4*, *TGFA* and *ESR1*. *ERBB4* is required for the differentiation of mouse mammary epithelial cells during pregnancy and promotes differentiation of murine and human mammary epithelial cells in cell culture [44]. However, other animal models demonstrated that *TGFA* promotes epithelium growth in mammary glands by binding to the EGF receptor, activating its kinase cell signaling. Meanwhile, luminal cells perform essential functions in milk synthesis and secretion (Fig. 3G and H). *ACACA*, *FASN* and *ACLY* are key genes to improving the lactation function of luminal epithelial cells. *ACACA* and *FASN* are two critical genes required for fatty acid synthesis in milk, mainly acting in the elongation of the fatty acid chain [45, 46]. *ACLY* converts cytoplasmic citrate to acetyl-CoA and oxaloacetate and catalyzes the first step of the de novo lipogenesis pathway [47]. Epithelial cell maturity is marked by higher expression levels of genes associated with casein synthesis [48, 49], such as *CSN1S1* and *CSN2*, whose expression are detected at single cell and spatial levels.

At the onset of lactation (L0), the proliferation of mammary cells, except adipocytes and precursor cells, is strongly affected by the cytokine-type L-R pairs, CXCL12-ITGB1. The team of Ryota Kawahara proved that the phenotype of ITGB1-KO mice is embryonic lethal [50]. In L0, the epithelial, myoepithelial and immune cells all promote growth, proliferation, migration of endothelial cells and vascular network formation through PDGFC-KDR pathway (Fig. 5 and Fig. S2) [51]. Fibroblasts regulate endothelial cell differentiation and promote angiogenesis by secreting VEGFC ligand, one of the strongest modulators of angiogenesis [52], via FLT1, FLT4, LYVE1, ITGA9 and ITGB1 receptors [53–57]. These L-R pathways jointly promote angiogenesis and ensure the transportation of nutrients required for lactation [58, 59].

Epithelial cells initiate lactation (L0) through hormone signals. As lactation progresses, epithelial cells actively synthesize milk to meet nutritional requirements for newborns. After fulfilling lactation mission, epithelial cells undergo programed cell death (L20). Cell–cell interaction analysis indicated that epithelial cells promoted the proliferation and differentiation of immune cells by secreting IL34 ligand and binding to CSF1R surface receptor [60, 61]. This suggests that epithelial cells can be actively cleared by immune cells to eliminate dead cells under high intensity lactation. This biological process may be an important mechanism for maintaining homeostasis of the mammary gland. It has been established that immune cells including neutrophils, macrophages and lymphocytes are present in milk [62]. Numerous data on animal studies have shown that maternal immune cells can be transferred to newborns through milk. Langel et al. [63] suggests that these maternal immune cells contribute to the maturation of the innate immune system in the offspring. There are abundant macrophages in colostrum. With the lactation process, the number of macrophages in mature milk decreases [64, 65]. Importantly, these macrophages often elicit significant and non-homeostatic inflammatory responses. In 2020, Zimmermann and Macpherson [66] demonstrated that T-regulatory cells (Treg) can be transmitted to the offspring via milk, and persist for a long time in a mechanism that provides microbial and pathogenic resistance to the offspring. These results suggest that Treg in milk can help newborns build an early immune barrier.

Our data indicates that the adipocytes area accounts for less than 0.5% during lactation (L0 and L20). This results from the dedifferentiation mechanism of adipocytes. To assure survival in the extremely stressful living environment, the adipocyte maintains a poorly differentiated state with high proliferative capacity, which may be driven by hormone inhibition of adipocyte differentiation (the canonical insulin receptor signaling pathway and AMPK signaling pathway). In the two pathways, three key genes (*ZFP36L1*, *FOXO1* and *LPIN1*) inhibit the differentiation of adipocytes. *ZFP36L1* inhibits intracellular fat synthesis [67], while *FOXO1* gene inhibits adipocyte differentiation [68]. Previous studies have shown that *LPIN1* expression is higher in preadipocytes than in mature adipocytes [69, 70]. Similarly, *LPIN1* displays a higher expression in L0 and L20 rather than G90. At mature milk stage (L20), the cell–cell interactions show that the cytokine ligands secreted by endothelial cells (CCL5, CCL21) and myoepithelial cells (CCL2) activate the adipocyte surface receptor ACKR4 (Fig. 5C and Fig. S2). After receiving CCL21 signals from other cells, adipocytes reportedly activate ACKR4 to remove chemokines and evade phagocytosis by inflammatory cells [71–73]. A previous study confirms that the absence of FGFR1 gene leads to a delay in mammary gland development, with a short-term decrease in cell proliferation [74]. This may indicate that the autocrine FGF10-FGFR1 pathway is the required for proliferation potential of adipocytes during L20 (Fig. 5 and Fig. S2). Besides, adipocytes inhibit apoptosis via the ADIPOQ-ADIPOR2 pathway [6, 75]. Overall, adipocytes are actively mobilizing their growth potential in the period of physiological lactation degradation and preparing for remodeling.

Previous research has confirmed that the mammary gland undergoes the epithelial programmed cell death during lactational involution [76]. Our latest work anatomizes this process in more detail. Epithelial cell populations first stop proliferating (Rap1 signaling pathway), then undergo programmed cell death (NF-kappa B signaling pathway and IL-17 signaling pathway), and the last surviving cells dedifferentiate into mesenchymal stem cells (*BMPR1A*, *TGFBR3* and *WWTR1*). In detail, the Rap1 signaling pathway plays an important role in cell proliferation [77], while the NF-kappa B signaling pathway and IL-17 signaling pathway drive apoptosis in epithelial cells [78, 79]. Non-apoptotic epithelial cells are dedifferentiated to mesenchymal cells as “seeds” for subsequent lactation cycles. The transforming growth factor beta receptor 3 (*TGFBR3*) and WW domain containing transcription regulator 1 (*WWTR1*) induce the transformation of epithelial cells into mesenchymal cells [80, 81]. Additionally, the bone morphogenetic protein receptor type 1A (*BMPR1A*) plays an important role in maintaining the undifferentiated state of mammary epithelial cells [82]. Volume reduction is a critical feature in mammary gland involution after weaning. To maintain gland homeostasis, immune cells are required to clear the programmed dead cells [81]. Surprisingly, these cells seem to be actively cleared by immune cells. This conclusion is based on the results of the gene transcription dynamics and cell–cell interaction analysis. At this stage, mammary cells signal the demand for proliferation and differentiation to immune cells via IL34-CSF1R pairs [31, 82]. Similarly, epithelial cells stimulate myoepithelial cell growth through the growth factor type ligand-receptor (TGFA-EGFR) pathway during early natural involution (Fig. 5 and Fig. S2) [79]. We speculate that this dynamic transcription profile is related to epithelial cells role in ejection of milk in the acini through the myoepithelial cell contraction to reduce the inflammatory reaction in the mammary gland. In 7 d post natural involution (PI7), TGFA ligand activates the receptor ERBB4 on the surface of adjacent epithelial cells to maintain proliferation, so that the epithelial cells persist in an extremely narrow living space [32]. This molecular mechanism maintains the lactation potential of the mammary gland and guarantees the next lactation cycle.

Adipocytes area increase by 22.85 percentage points at PI7 versus L20. The regulation of lipid storage and lipid biosynthetic process leads to extracellular lipid transport into the cytoplasm and fusion with other lipid droplets to increase the size of fat adipocytes, which is one of the important ways to hypertrophy [83]. This is consistent with a report from Zwick et al. [84] that the regulatory mechanism of adipocyte hypertrophy and reoccupation during mammary gland remodeling in mice. In remodeling of mammary glands, the regulation of lipid storage term (*CD36*), the response to fatty acid term (*LPL*), and the triglyceride metabolic process term (*ABCA1*) are activated. The *ABCA1* can regulate adipocyte lipid metabolism by adjusting the lipid content of adipose tissue, glucose tolerance, and insulin sensitivity [85, 86]. *CD36* is a scavenger receptor that plays a role in adipose energy storage [87]. The *LPL* gene mainly participates in the uptake of lipids by adipocytes, and is a critical regulatory factor for lipid accumulation in adipocytes, as well as a marker for adipocyte differentiation [88, 89]. In summary, the lineage trajectory analysis revealed that adipocytes underwent dedifferentiation, proliferation and redifferentiation from late pregnancy to natural involution (PI7) [6, 9]. Adipocytes frequently received cytokine signals from other cell types (Fig. 5D and Fig. S2). This phenotype was closely related to the active genes involved in proliferation and differentiation. In PI2, the surface cytokine receptor, ACKR4, participated in adipocyte chemokine’s clearance [77, 78, 90]. Notably, different from mature milk stage, the autocrine pathways maintain adipocyte proliferation through FGF1-EGFR and IGFBP4-LRP6 pairs at PI2, rather than FGF10-FGFR1 pairs (Fig. 5 and Fig. S2) [79, 80]. The above molecular mechanisms maintain the lactation potential of the mammary gland and guarantee the next lactation cycle.

## Conclusion

Taken together, this dataset is the largest cell transcriptomic profile of porcine mammary gland across development to date. Herein, we reveal the internal factors of proliferation, differentiation and apoptosis of epithelial cells and adipocytes across five development stages. A vital finding was that epithelial cells are converted into mesenchymal stem cells during the remodeling process. Of note, our data annotated adipocytes in the porcine mammary glands, and clarified the molecular mechanism of dedifferentiation, proliferation and redifferentiation in adipocytes, from late pregnancy to natural involution. Overall, our data provide novel and fundamental insights into the mammary gland development.

### Supplementary Information

**Additional file 1: Table S1. **The phenotype data of adipocytes in the mammary gland (mean ± SD). **Table S2.** The information of sequencing statistics and cell statistics. **Table S3.** Maker genes used for cell type annotation. **Table S4.** Fraction of each cell type in each developmental stage. **Table S5.** Statistics of the number of differentially expressed genes identified in two adjacent periods in each cell type. **Fig. S1.** The diagrams showed the nFeatures, nCounts, mitochondrial percent and ribosomal percent of each sample before or after quality control. **Fig. S2.** The typical L-R interactions predicted by iTALK between any two cell types. **Fig. S3.** Deconvolution of ST data based on snRNA-seq data. **Fig. S4.** GO annotation and KEGG pathway analysis of upregulated DEGs identified in adjacent developmental stages. **Fig. S5.** The common significant GO terms and KEGG pathways identified in adjacent developmental stages. **Fig. S6.** Venn graph of the numbers of shared and unique significant GO term sets or KEGG pathway sets in different cell types (adipocytes, epithelial, fibroblasts, endothelial, myoepithelial, immune and precursor cells; indicated with different color) in the mammary gland of five devel-opmental stages (L0 vs. G90, L20 vs. L0, PI2 vs. L20, and PI7 vs. PI2 groups).

## Abbreviations

DEGs: Differentially expressed genes
GEO: Gene Expression Omnibus
GO: Gene Ontology
H&E: Hematoxylin and Eosin
KEGG: Kyoto Encyclopedia of Genes and Genomes
L: Ligand
L-R: Ligand-receptor
PCA: Principal component analysis
R: Receptor
scRNA-seq: Single-cell RNA sequencing
snRNA-seq: Single-nucleus RNA-seq
ST: Spatial transcriptomics
UMAP: Uniform manifold approximation and projection
