# Supplementary material for: A cell transcriptomic profile provides insights into adipocytes of porcine mammary gland across development
Source: J Anim Sci Biotechnol. 2023 Oct 8;14:126. doi: 10.1186/s40104-023-00926-0 (PMC10560433; doi:10.1186/s40104-023-00926-0)
Supplement: Supplementary file 1 — Additional file 1: Table S1. The phenotype data of adipocytes in the mammary gland (mean ± SD). Table S2. The information of sequencing statistics and cell statistics. Table S3. Maker genes used for cell type annotation. Table S4. Fraction of each cell type in each developmental stage. Table S5. Statistics of the number of differentially expressed genes identified in two adjacent periods in each cell type. Fig. S1. The diagrams showed the nFeatures, nCounts, mitochondrial percent and ribosomal percent of each sample before or after quality control. Fig. S2. The typical L-R interactions predicted by iTALK between any two cell types. Fig. S3. Deconvolution of ST data based on snRNA-seq data. Fig. S4. GO annotation and KEGG pathway analysis of upregulated DEGs identified in adjacent developmental stages. Fig. S5. The common significant GO terms and KEGG pathways identified in adjacent developmental stages. Fig. S6. Venn graph of the numbers of shared and unique significant GO term sets or KEGG pathway sets in different cell types (adipocytes, epithelial, fibroblasts, endothelial, myoepithelial, immune and precursor cells; indicated with different color) in the mammary gland of five devel-opmental stages (L0 vs. G90, L20 vs. L0, PI2 vs. L20, and PI7 vs. PI2 groups). [file 40104_2023_926_MOESM1_ESM.docx]

**Table S1** The phenotype data of adipocytes in the mammary gland (mean ± SD)

|  | **G90** | **L0** | **L20** | **PI2** | **PI7** | ***P-*value** |
| --- | --- | --- | --- | --- | --- | --- |
| The area of single adipocyte, μm^2^ | 2,514.92 ± 132.17^a^ | 398.49 ± 36.97^c^ | 554.94 ± 40.29^c^ | 1,326.67 ± 89.65^b^ | 2,461.14 ± 118.37^a^ | ＜0.05 |
| The CV of the area of single adipocyte, % | 5.26 | 9.28 | 7.26 | 6.76 | 4.81 |  |
| The diameter of single adipocyte, μm | 54.06 ± 6.33^a^ | 25.16 ± 2.83^c^ | 28.23 ± 3.58^c^ | 39.53 ± 5.33^b^ | 53.99 ± 6.14^a^ | ＜0.05 |
| The CV of the diameter of single adipocyte, % | 11.71 | 11.25 | 12.70 | 13.50 | 11.37 |  |
| The area of adipocytes , % | 27.60 | 0.38 | 0.32 | 16.26 | 23.17 | - |

^a–c^*P* < 0.05 in the row

**Table S2** The information of sequencing statistics and cell statistics

| **Sample** | **Estimated Number of Cells** | **Sequencing Depth** | **Median UMI per Cell** | **Saturation , %** | **Median Genes per Cell** |
| --- | --- | --- | --- | --- | --- |
| G90_LY1 | 11,484 | 61,186 | 3,673 | 64.70 | 1,923 |
| G90_LY2 | 18,697 | 32,805 | 2,986 | 55.79 | 1,624 |
| L0_LY1 | 11,509 | 65,658 | 2,349 | 77.91 | 1,244 |
| L0_LY2 | 9,447 | 73,638 | 2,489 | 77.10 | 1,310 |
| L20_LY1 | 13,007 | 79,460 | 1,787 | 59.20 | 1,074 |
| L20_LY2 | 15,338 | 59,450 | 3,109 | 69.13 | 1,599 |
| PI2_LY1 | 12,463 | 53,431 | 1,922 | 75.83 | 1,159 |
| PI2_LY2 | 9,512 | 60,199 | 1,728 | 71.36 | 971 |
| PI7_LY1 | 16,695 | 61,594 | 2,326 | 72.69 | 1,355 |
| PI7_LY2 | 10,081 | 62,494 | 3,046 | 58.23 | 1,688 |

**Table S3** Maker genes used for cell type annotation

| **Cell type** | **Marker genes** |
| --- | --- |
| Epithelial cells | *CSN2,* *PGR*, *ESR1*, *PRLR*, *LALBA* [1]; *CSN3* [2]; *EPCAM* [3]; *KRT18* [4]; *ELF5* [5]; *TPM2* [6]; *CLDN4* [7] |
| Adipocyte | *ADIPOQ* [8]; *PPARG* [9]; *CD36* [10] |
| Endothelial cells | *MMRN1* [11]; *FLT4* [12]; *PARD6G*, *JAM2* [13]; *ENG*, *PLVAP* [14]; *GPNM8*, *DAB2* [15]; *CDSD* [16] |
| Myoepithelial cells | *MYH11*, *ACAT2*, *MYLK*, *COL3A1*, *COL1A2*, *COL1A1* [2]; *DCN*, *POSTN* [17]; *COL6A3* [18] |
| Immune cells | *CD136*, *PTPRC* [2]; *ZEB2* [19]; *BANK1* [20]; *CST3* [21]; *COX2* [22]; *CD3E* [23]; *KLRK1* [24]; *ATP6* [25] |
| Precursor cells | *IL1R1* [26]; *DLG2*, *LARGE1*, *NAV2* [27]; *ZNF710* [28]; *SERINC5* [29]; *RABEP1* [30]; *MSI2* [31]; *WWOX* [32]; *LIFR* [33]; *KIF15*, *TPX2*, *SMC4* [34]; *TOP2A* [35] |
| Macrophages | *TNFAIP6* [36]; *CD63* [37]; *FCER1G* [38]; *CD163* [39]; *MSR1* [40]; *CXCR4* [41]; *CCL4* [42]; *CD24* [43]; *CD14* [44]; *TREM2* [45]; *AIF1* [18]; *SLC7A7*, *S100A8* [46] |
| Monocytes | *CLEC7A* [31]; *CORO1A* [25]; *CCRL2* [47]; *CSF3R* [48]; *CD69* [49]; *IL1R2* [50]; *S100A11* [51]; *MMP8* [52] |
| T cells | *CD4*, *CD8A* [53]; *CD8B* [54]; *GNLY* [55]; *CST7* [56]; *ETS1* [57]; *GZMH* [58]; *CD2* [59] |

**Table S4** Fraction of each cell type in each developmental stage

| **Cell type** | **G90** | **L0** | **L20** | **PI2** | **PI7** |
| --- | --- | --- | --- | --- | --- |
| Immune cells | 20.44% | 9.67% | 15.63% | 8.44% | 31.25% |
| Fibroblasts cells | 6.77% | 1.90% | 4.68% | 12.50% | 10.37% |
| Myoepithelial cells | 6.38% | 1.49% | 2.02% | 7.99% | 8.94% |
| Precursor cells | 18.55% | 11.44% | 10.16% | 4.49% | 12.55% |
| Epithelial cells | 37.97% | 72.43% | 63.07% | 49.37% | 19.37% |
| Endothelial cells | 9.54% | 2.98% | 4.35% | 17.01% | 17.32% |
| Adipocytes | 0.34% | 0.10% | 0.09% | 0.21% | 0.20% |

**Table S5** Statistics of the number of differentially expressed genes identified in two adjacent periods in each cell type

|  | **L0 vs. G90** | | | **L20 vs. L0** | | | **PI2 vs. L20** | | | **PI7 vs. PI2** | | |
| --- | --- | --- | --- | --- | --- | --- | --- | --- | --- | --- | --- | --- |
|  | **Up** | **Down** | **Total** | **Up** | **Down** | **Total** | **Up** | **Down** | **Total** | **Up** | **Down** | **Total** |
| Epithelial cells | 1,082 | 1,739 | 2,821 | 1,352 | 1,261 | 2,613 | 750 | 646 | 1,396 | 908 | 758 | 1,666 |
| Precursor cells | 616 | 1,205 | 1,821 | 595 | 740 | 1,335 | 887 | 601 | 1,488 | 718 | 742 | 1,460 |
| Immune cells | 850 | 1,340 | 2,190 | 1,478 | 1,164 | 2,642 | 795 | 989 | 1,784 | 1,038 | 644 | 1,682 |
| Fibroblasts cells | 329 | 1,379 | 1,708 | 1,186 | 390 | 1,576 | 901 | 1,035 | 1,936 | 732 | 677 | 1,409 |
| Endothelial cells | 414 | 783 | 1,197 | 842 | 603 | 1,445 | 1,034 | 810 | 1,844 | 810 | 765 | 1,575 |
| Myoepithelial cells | 243 | 1,328 | 1,571 | 870 | 424 | 1,294 | 821 | 754 | 1,575 | 780 | 366 | 1,146 |
| Adipocytes | 228 | 129 | 357 | 1,627 | 271 | 1,898 | 1,887 | 1,010 | 2,897 | 139 | 1,193 | 1,332 |
| Range |  |  | 2,464 |  |  | 1,348 |  |  | 1,501 |  |  | 536 |


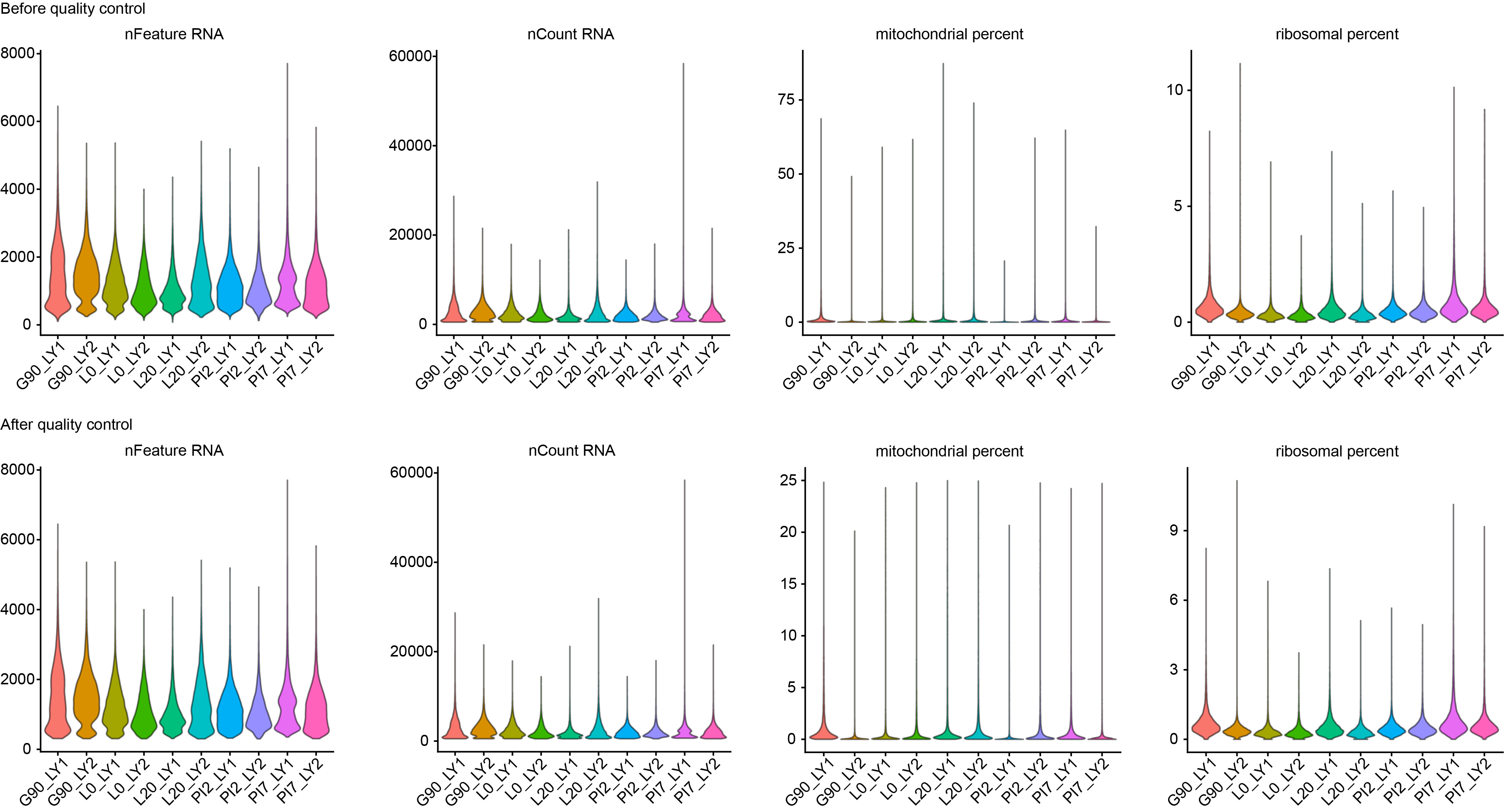


**Fig. S1** The diagrams showed the nFeatures, nCounts, mitochondrial percent and ribosomal percent of each sample before or after quality control





**Fig. S2** The typical L-R interactions predicted by iTALK between any two cell types


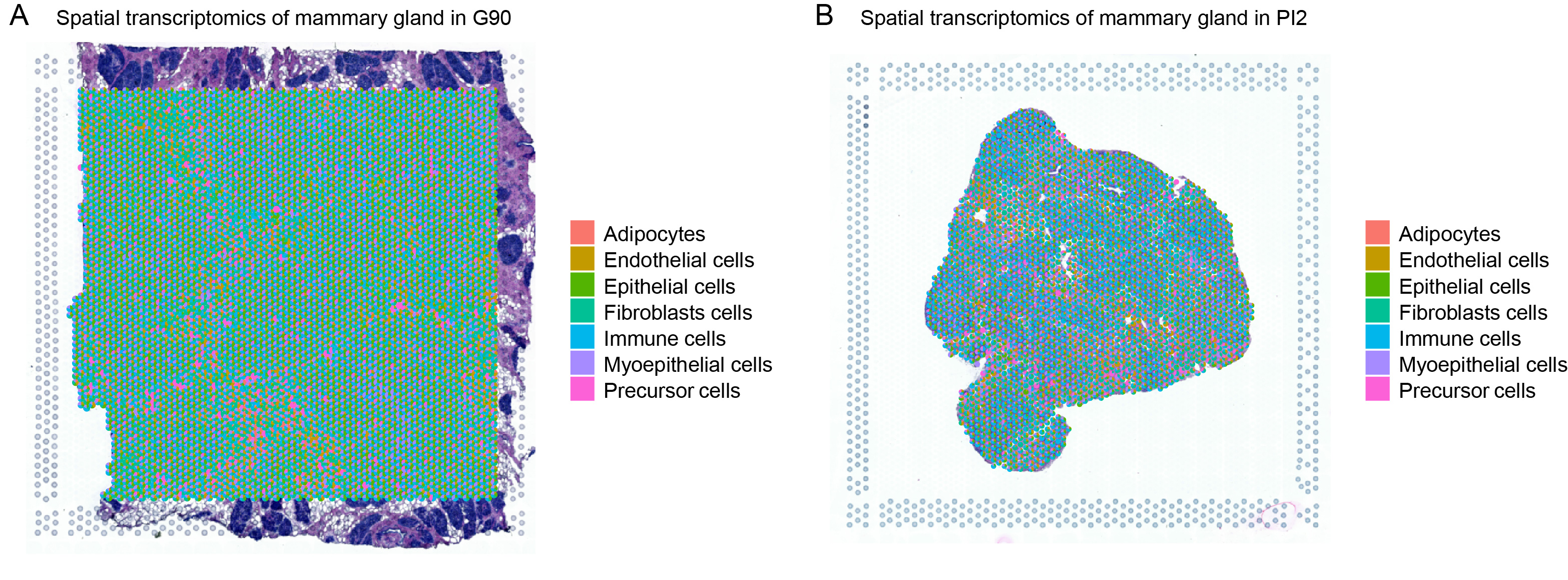


**Fig. S3** Deconvolution of ST data based on snRNA-seq data. Each pie chart represented the contribution of the cell types from the single-nuclei reference data set to the transcriptomic signature of each spot in the mammary gland. Only cell types contributing to at least 10% of the spot signature were displayed





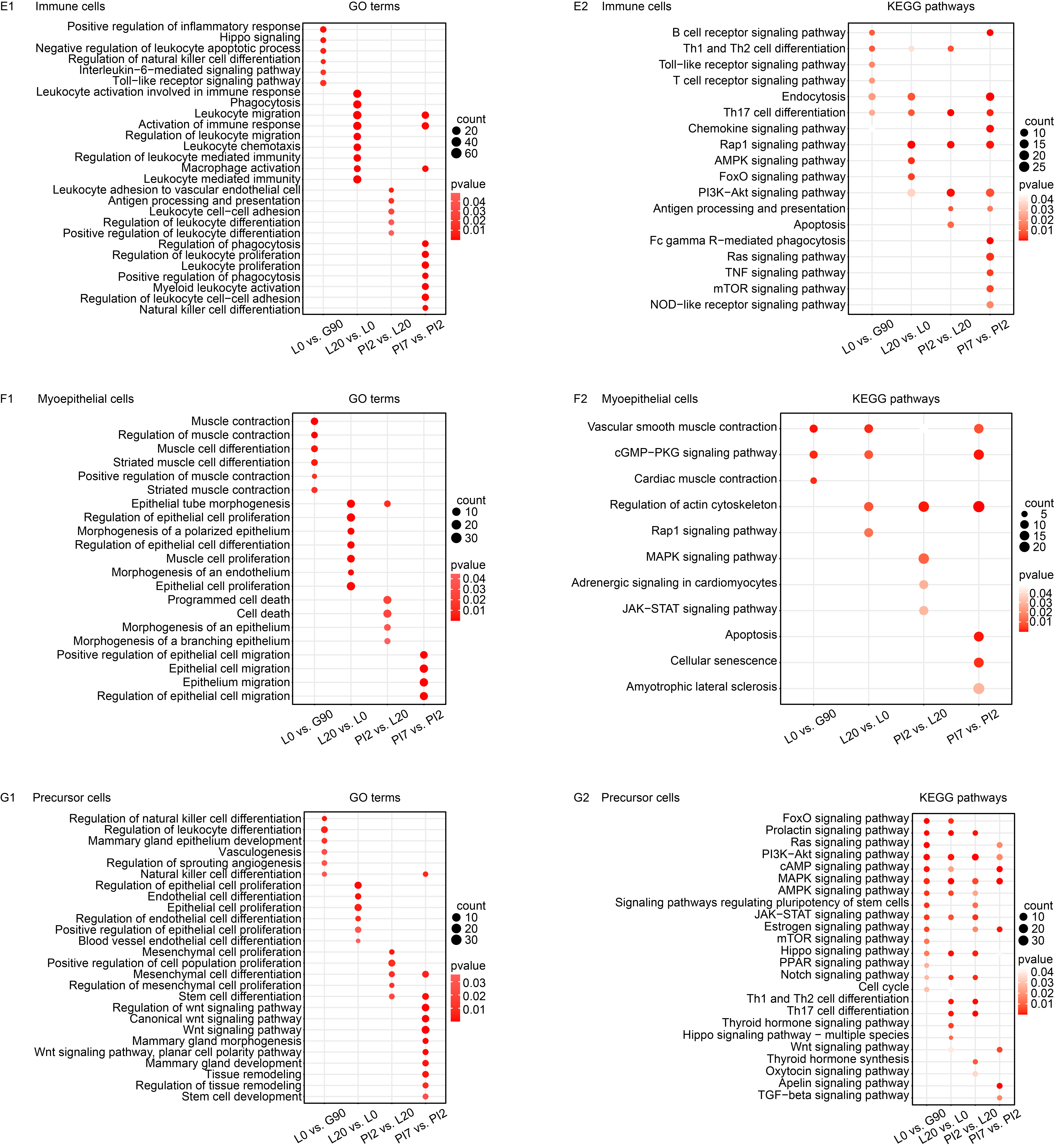


**Fig. S4** GO annotation and KEGG pathway analysis of upregulated DEGs identified in adjacent developmental stages





**Fig. S5** The common significant GO terms and KEGG pathways identified in adjacent developmental stages





**Fig. S6** Venn graph of the numbers of shared and unique significant GO term sets or KEGG pathway sets in different cell types (adipocytes, epithelial, fibroblasts, endothelial, myoepithelial, immune and precursor cells; indicated with different color) in the mammary gland of five devel-opmental stages (L0 vs. G90, L20 vs. L0, PI2 vs. L20, and PI7 vs. PI2 groups). The overlapping part represents the shared GO term or KEGG pathway; non-overlapping part represents the specific GO term or KEGG pathway of the given depth; and the number means the corresponding amount of GO term or KEGG pathway

**References**

1. Bach K, Pensa S, Grzelak M, Hadfield J, Adams DJ, Marioni JC, et al. Differentiation dynamics of mammary epithelial cells revealed by single-cell RNA sequencing. Nat Commun. 2017;8:2128. https://doi.org/10.1038/s41467-017-02001-5.

2. Li CM, Shapiro H, Tsiobikas C, Selfors LM, Chen H, Rosenbluth J, et al. Aging-associated alterations in mammary epithelia and stroma revealed by single-cell RNA sequencing. Cell Rep. 2020;33(13):108566. https://doi.org/10.1016/j.celrep.2020.108566.

3. Lourenco AR, Ban Y, Crowley MJ, Lee SB, Ramchandani D, Du W, et al. Differential contributions of pre- and post-EMT tumor cells in breast cancer metastasis. Cancer Res. 2020;80(2):163–9. https://doi.org/10.1158/0008-5472.Can-19-1427.

4. Valdés-Mora F, Salomon R, Gloss BS, Law AMK, Venhuizen J, Castillo L, et al. Single-cell transcriptomics reveals involution mimicry during the specification of the basal breast cancer subtype. Cell Rep. 2021;35(2):108945. https://doi.org/10.1016/j.celrep.2021.108945.

5. Guo M, Wang H, Potter SS, Whitsett JA, Xu Y. SINCERA: A pipeline for single-cell RNA-Seq profiling analysis. PLoS Comput Biol. 2015;11(11):e1004575. https://doi.org/10.1371/journal.pcbi.1004575.

6. Bannier-Hélaouët M, Post Y, Korving J, Trani Bustos M, Gehart H, Begthel H, et al. Exploring the human lacrimal gland using organoids and single-cell sequencing. Cell Stem Cell. 2021;28(7):1221–32.e1227. https://doi.org/10.1016/j.stem.2021.02.024.

7. Lu TM, Houghton S, Magdeldin T, Durán JGB, Minotti AP, Snead A, et al. Pluripotent stem cell-derived epithelium misidentified as brain microvascular endothelium requires ETS factors to acquire vascular fate. Proc Natl Acad Sci U S A. 2021;118(8). https://doi.org/10.1073/pnas.2016950118.

8. Bäckdahl J, Franzén L, Massier L, Li Q, Jalkanen J, Gao H, et al. Spatial mapping reveals human adipocyte subpopulations with distinct sensitivities to insulin. Cell Metab. 2021;33(9):1869–82.e1866. https://doi.org/10.1016/j.cmet.2021.07.018.

9. Zhong L, Yao L, Tower RJ, Wei Y, Miao Z, Park J, et al. Single cell transcriptomics identifies a unique adipose lineage cell population that regulates bone marrow environment. Elife. 2020;9. https://doi.org/10.7554/eLife.54695.

10. Vijay J, Gauthier MF, Biswell RL, Louiselle DA, Johnston JJ, Cheung WA, et al. Single-cell analysis of human adipose tissue identifies depot and disease specific cell types. Nat Metab. 2020;2(1):97–109. https://doi.org/10.1038/s42255-019-0152-6.

11. Petitprez F, de Reyniès A, Keung EZ, Chen TW, Sun CM, Calderaro J, et al. B cells are associated with survival and immunotherapy response in sarcoma. Nature. 2020;577(7791):556–60. https://doi.org/10.1038/s41586-019-1906-8.

12. van Zyl T, Yan W, McAdams A, Peng YR, Shekhar K, Regev A, et al. Cell atlas of aqueous humor outflow pathways in eyes of humans and four model species provides insight into glaucoma pathogenesis. Proc Natl Acad Sci U S A. 2020;117(19):10339–49. https://doi.org/10.1073/pnas.2001250117.

13. Wu SZ, Roden DL, Wang C, Holliday H, Harvey K, Cazet AS, et al. Stromal cell diversity associated with immune evasion in human triple-negative breast cancer. Embo J. 2020;39(19):e104063. https://doi.org/10.15252/embj.2019104063.

14. Yuan X, Mei B, Zhang L, Zhang C, Zheng M, Liang H, et al. Enhanced penetration of exogenous EPCs into brains of APP/PS1 transgenic mice. Am J Transl Res. 2016;8(3):1460–70. PMID:27186272; PMCID:PMC4859631.

15. Guo FH, Guan YN, Guo JJ, Zhang LJ, Qiu JJ, Ji Y, et al. Single-cell transcriptome analysis reveals embryonic endothelial heterogeneity at spatiotemporal level and multifunctions of microRNA-126 in mice. Arterioscler Thromb Vasc Biol. 2022;42(3):326–42. https://doi.org/10.1161/atvbaha.121.317093.

16. Li B, Nie Z, Zhang D, Wu J, Peng B, Guo X, et al. Roles of circulating endothelial progenitor cells and endothelial cells in gastric carcinoma. Oncol Lett. 2018;15(1):324–30. https://doi.org/10.3892/ol.2017.7272.

17. Meng S, Lv J, Chanda PK, Owusu I, Chen K, Cooke JP. Reservoir of Fibroblasts Promotes Recovery from limb Ischemia. Circulation. 2020;142(17):1647–62. https://doi.org/10.1161/circulationaha.120.046872.

18. Galvani E, Mundra PA, Valpione S, Garcia-Martinez P, Smith M, Greenall J, et al. Stroma remodeling and reduced cell division define durable response to PD-1 blockade in melanoma. Nat Commun. 2020;11:853. https://doi.org/10.1038/s41467-020-14632-2.

19. Friedrich C, Taggenbrock R, Doucet-Ladevèze R, Golda G, Moenius R, Arampatzi P, et al. Effector differentiation downstream of lineage commitment in ILC1s is driven by Hobit across tissues. Nat Immunol. 2021;22(10):1256–67. https://doi.org/10.1038/s41590-021-01013-0.

20. He D, Mao A, Zheng CB, Kan H, Zhang K, Zhang Z, et al. Aortic heterogeneity across segments and under high fat/salt/glucose conditions at the single-cell level. Natl Sci Rev. 2020;7(5):881–96. https://doi.org/10.1093/nsr/nwaa038.

21. Park J, Shrestha R, Qiu C, Kondo A, Huang S, Werth M, et al. Single-cell transcriptomics of the mouse kidney reveals potential cellular targets of kidney disease. Science. 2018;360(6390):758–63. https://doi.org/10.1126/science.aar2131.

22. Matijevic N, Wu KK, Nidkarni N, Heiss G, Folsom AR. The ARIC carotid MRI study of blood cellular markers: an inverse association of monocyte myeloperoxidase content with peripheral arterial disease. Angiology. 2011;62(3):237–44. https://doi.org/10.1177/0003319710385336.

23. O'Flanagan CH, Campbell KR, Zhang AW, Kabeer F, Lim JLP, Biele J, et al. Dissociation of solid tumor tissues with cold active protease for single-cell RNA-seq minimizes conserved collagenase-associated stress responses. Genome Biol. 2019;20:210. https://doi.org/10.1186/s13059-019-1830-0.

24. Anand P, Guillaumet-Adkins A, Dimitrova V, Yun H, Drier Y, Sotudeh N, et al. Single-cell RNA-seq reveals developmental plasticity with coexisting oncogenic states and immune evasion programs in ETP-ALL. Blood. 2021;137(18):2463–80. https://doi.org/10.1182/blood.2019004547.

25. Young MD, Mitchell TJ, Vieira Braga FA, Tran MGB, Stewart BJ, Ferdinand JR, et al. Single-cell transcriptomes from human kidneys reveal the cellular identity of renal tumors. Science. 2018;361(6402):594-99. https://doi.org/10.1126/science.aat1699.

26. Hochane M, van den Berg PR, Fan X, Bérenger-Currias N, Adegeest E, Bialecka M, et al. Single-cell transcriptomics reveals gene expression dynamics of human fetal kidney development. PLoS Biol. 2019;17(2):e3000152. https://doi.org/10.1371/journal.pbio.3000152.

27. Zhou F, Li X, Wang W, Zhu P, Zhou J, He W, et al. Tracing haematopoietic stem cell formation at single-cell resolution. Nature. 2016;533(7604):487–92. https://doi.org/10.1038/nature17997.

28. Gao S, Yan L, Wang R, Li J, Yong J, Zhou X, et al. Tracing the temporal-spatial transcriptome landscapes of the human fetal digestive tract using single-cell RNA-sequencing. Nat Cell Biol. 2018;20(6):721–34. https://doi.org/10.1038/s41556-018-0105-4.

29. Petropoulos S, Edsgärd D, Reinius B, Deng Q, Panula SP, Codeluppi S, et al. Single-cell RNA-Seq reveals lineage and X chromosome dynamics in human preimplantation embryos. Cell. 2016;165(4):1012–26. https://doi.org/10.1016/j.cell.2016.03.023.

30. Grün D, Lyubimova A, Kester L, Wiebrands K, Basak O, Sasaki N, et al. Single-cell messenger RNA sequencing reveals rare intestinal cell types. Nature. 2015;525(7568):251–55. https://doi.org/10.1038/nature14966.

31. Giladi A, Paul F, Herzog Y, Lubling Y, Weiner A, Yofe I, et al. Single-cell characterization of haematopoietic progenitors and their trajectories in homeostasis and perturbed haematopoiesis. Nat Cell Biol. 2018;20(7):836–46. https://doi.org/10.1038/s41556-018-0121-4.

32. Karamitros D, Stoilova B, Aboukhalil Z, Hamey F, Reinisch A, Samitsch M, et al. Single-cell analysis reveals the continuum of human lympho-myeloid progenitor cells. Nat Immunol. 2018;19:85–97. https://doi.org/10.1038/s41590-017-0001-2.

33. Keshel SH, Soleimani M, Tavirani MR, Ebrahimi M, Raeisossadati R, Yasaei H, et al. Evaluation of unrestricted somatic stem cells as a feeder layer to support undifferentiated embryonic stem cells. Mol Reprod Dev. 2012;79(10):709–18. https://doi.org/10.1002/mrd.22079.

34. Zhong S, Zhang S, Fan X, Wu Q, Yan L, Dong J, et al. A single-cell RNA-seq survey of the developmental landscape of the human prefrontal cortex. Nature. 2018;555(7697):524–28. https://doi.org/10.1038/nature25980.

35. Pérez MJ, Ivanyuk D, Panagiotakopoulou V, Di Napoli G, Kalb S, Brunetti D, et al. Loss of function of the mitochondrial peptidase PITRM1 induces proteotoxic stress and Alzheimer's disease-like pathology in human cerebral organoids. Mol Psychiatry. 2021;26(10):5733–50. https://doi.org/10.1038/s41380-020-0807-4.

36. Walter JM, Ren Z, Yacoub T, Reyfman PA, Shah RD, Abdala-Valencia H, et al. Multidimensional assessment of the host response in mechanically ventilated patients with suspected pneumonia. Am J Respir Crit Care Med. 2019;199(10):1225–37. https://doi.org/10.1164/rccm.201804-0650OC.

37. Jaitin DA, Adlung L, Thaiss CA, Weiner A, Li B, Descamps H, et al. Lipid-associated macrophages control metabolic homeostasis in a Trem2-dependent manner. Cell. 2019;178(3):686–98.e614. https://doi.org/10.1016/j.cell.2019.05.054.

38. Iqbal F, Lupieri A, Aikawa M, Aikawa E. Harnessing single-Cell RNA sequencing to better understand how diseased cells behave the way they do in cardiovascular disease. Arterioscler Thromb Vasc Biol. 2021;41(2):585–600. https://doi.org/10.1161/atvbaha.120.314776.

39. Lago N, Kaufmann FN, Negro-Demontel ML, Alí-Ruiz D, Ghisleni G, Rego N, et al. CD300f immunoreceptor is associated with major depressive disorder and decreased microglial metabolic fitness. Proc Natl Acad Sci U S A. 2020;117(12):6651–62. https://doi.org/10.1073/pnas.1911816117.

40. Theocharidis G, Baltzis D, Roustit M, Tellechea A, Dangwal S, Khetani RS, et al. Integrated skin transcriptomics and serum multiplex assays reveal novel mechanisms of wound healing in diabetic foot ulcers. Diabetes. 2020;69(10):2157–69. https://doi.org/10.2337/db20-0188.

41. Farmer DT, Nathan S, Finley JK, Shengyang Yu K, Emmerson E, Byrnes LE, et al. Defining epithelial cell dynamics and lineage relationships in the developing lacrimal gland. Development. 2017;144(13):2517–28. https://doi.org/10.1242/dev.150789.

42. Pedroza AJ, Tashima Y, Shad R, Cheng P, Wirka R, Churovich S, et al. Single-cell transcriptomic profiling of vascular smooth muscle cell phenotype modulation in marfan syndrome aortic aneurysm. Arterioscler Thromb Vasc Biol. 2020;40(9):2195–211. https://doi.org/10.1161/atvbaha.120.314670.

43. Wang Y, Chaffee TS, LaRue RS, Huggins DN, Witschen PM, Ibrahim AM, et al. Tissue-resident macrophages promote extracellular matrix homeostasis in the mammary gland stroma of nulliparous mice. Elife. 2020;9. https://doi.org/10.7554/eLife.57438.

44. Daemen S, Schilling JD. The Interplay between tissue niche and macrophage cellular metabolism in obesity. Front Immunol. 2019;10:3133. https://doi.org/10.3389/fimmu.2019.03133.

45. Shen Q, Wang Y, Chen J, Ma L, Huang X, Tang SCW, et al. Single-cell RNA sequencing reveals the immunological profiles of renal allograft rejection in mice. Front Immunol. 2021;12:693608. https://doi.org/10.3389/fimmu.2021.693608.

46. Wang X, Yang L, Wang YC, Xu ZR, Feng Y, Zhang J, et al. Comparative analysis of cell lineage differentiation during hepatogenesis in humans and mice at the single-cell transcriptome level. Cell Res. 2020;30(12):1109–26. https://doi.org/10.1038/s41422-020-0378-6.

47. Matthew DY, Thomas JM, Felipe AVB, Maxine GBT, Benjamin JS, John RF, et al. Single-cell transcriptomes from human kidneys reveal the cellular identity of renal tumors. Science. 2018;361(6402):594–99. https://doi.org/10.1126/science.aat1699.

48. Liu M, Tayob N, Penter L, Sellars M, Tarren A, Chea V, et al. Improved T-cell immunity following neoadjuvant chemotherapy in ovarian cancer. Clin Cancer Res. 2022;28(15):3356–66. https://doi.org/10.1158/1078-0432.Ccr-21-2834.

49. Bischoff P, Trinks A, Obermayer B, Pett JP, Wiederspahn J, Uhlitz F, et al. Single-cell RNA sequencing reveals distinct tumor microenvironmental patterns in lung adenocarcinoma. Oncogene. 2021;40(50):6748–58. https://doi.org/10.1038/s41388-021-02054-3.

50. Valenzi E, Bulik M, Tabib T, Morse C, Sembrat J, Trejo Bittar H, et al. Single-cell analysis reveals fibroblast heterogeneity and myofibroblasts in systemic sclerosis-associated interstitial lung disease. Ann Rheum Dis. 2019;78(10):1379–87. https://doi.org/10.1136/annrheumdis-2018-214865.

51. Müller S, Kohanbash G, Liu SJ, Alvarado B, Carrera D, Bhaduri A, et al. Single-cell profiling of human gliomas reveals macrophage ontogeny as a basis for regional differences in macrophage activation in the tumor microenvironment. Genome Biol. 2017;18:234. https://doi.org/10.1186/s13059-017-1362-4.

52. Gross-Vered M, Trzebanski S, Shemer A, Bernshtein B, Curato C, Stelzer G, et al. Defining murine monocyte differentiation into colonic and ileal macrophages. Elife. 2020;9. https://doi.org/10.7554/eLife.49998.

53. Chen T, Delano MJ, Chen K, Sperry JL, Namas RA, Lamparello AJ, et al. A road map from single-cell transcriptome to patient classification for the immune response to trauma. JCI Insight. 2021;6(2). https://doi.org/10.1172/jci.insight.145108.

54. Lin CJ, Hunkins BM, Roth RA, Lin CY, Wagenseil JE, Mecham RP. Vascular smooth muscle cell subpopulations and neointimal formation in mouse models of elastin insufficiency. Arterioscler Thromb Vasc Biol. 2021;41(12):2890–905. https://doi.org/10.1161/atvbaha.120.315681.

55. Zhou W, Yu M, Mao X, Pan H, Tang X, Wang J, et al. Landscape of the peripheral immune response induced by local microwave ablation in patients with breast cancer. Adv Sci (Weinh). 2022;9(17):e2200033. https://doi.org/10.1002/advs.202200033.

56. Liu SQ, Gao ZJ, Wu J, Zheng HM, Li B, Sun S, et al. Single-cell and spatially resolved analysis uncovers cell heterogeneity of breast cancer. J Hematol Oncol. 2022;15(1):19. https://doi.org/10.1186/s13045-022-01236-0.

57. Nyquist SK, Gao P, Haining TKJ, Retchin MR, Golan Y, Drake RS, et al. Cellular and transcriptional diversity over the course of human lactation. Proc Natl Acad Sci U S A. 2022;119(15):e2121720119. https://doi.org/10.1073/pnas.2121720119.

58. Vázquez-Jiménez A, Avila-Ponce De León UE, Matadamas-Guzman M, Muciño-Olmos EA, Martínez-López YE, Escobedo-Tapia T, et al. On deep landscape exploration of COVID-19 patients cells and severity markers. Front Immunol. 2021;12:705646. https://doi.org/10.3389/fimmu.2021.705646.

59. Hu Y, Hu Y, Xiao Y, Wen F, Zhang S, Liang D, et al. Genetic landscape and autoimmunity of monocytes in developing Vogt-Koyanagi-Harada disease. Proc Natl Acad Sci U S A. 2020;117(41):25712–21. https://doi.org/10.1073/pnas.2002476117.
